# Increased Expression of Chemerin in Squamous Esophageal Cancer Myofibroblasts and Role in Recruitment of Mesenchymal Stromal Cells

**DOI:** 10.1371/journal.pone.0104877

**Published:** 2014-08-15

**Authors:** J. Dinesh Kumar, Chris Holmberg, Sandhir Kandola, Islay Steele, Peter Hegyi, Laszlo Tiszlavicz, Rosalind Jenkins, Robert J. Beynon, David Peeney, Olivier T. Giger, Ahlam Alqahtani, Timothy C. Wang, Trevor T. Charvat, Mark Penfold, Graham J. Dockray, Andrea Varro

**Affiliations:** 1 Department of Cell and Molecular Physiology, University of Liverpool, Liverpool, United Kingdom; 2 Department of Medicine and Surgery, University of Szeged, Szeged, Hungary; 3 Department of Pharmacology, Institute of Translational Medicine, University of Liverpool, Liverpool, United Kingdom; 4 Department of Pathology, University of Szeged, Szeged, Hungary; 5 Institute of Integrative Biology, University of Liverpool, Liverpool, United Kingdom; 6 ChemoCentryx, California, United States of America; 7 Department of Medicine, Columbia University Medical Center, New York, United States of America; University of South Alabama Mitchell Cancer Institute, United States of America

## Abstract

Stromal cells such as myofibroblasts influence tumor progression. The mechanisms are unclear but may involve effects on both tumor cells and recruitment of bone marrow-derived mesenchymal stromal cells (MSCs) which then colonize tumors. Using iTRAQ and LC-MS/MS we identified the adipokine, chemerin, as overexpressed in esophageal squamous cancer associated myofibroblasts (CAMs) compared with adjacent tissue myofibroblasts (ATMs). The chemerin receptor, ChemR23, is expressed by MSCs. Conditioned media (CM) from CAMs significantly increased MSC cell migration compared to ATM-CM; the action of CAM-CM was significantly reduced by chemerin-neutralising antibody, pretreatment of CAMs with chemerin siRNA, pretreatment of MSCs with ChemR23 siRNA, and by a ChemR23 receptor antagonist, CCX832. Stimulation of MSCs by chemerin increased phosphorylation of p42/44, p38 and JNK-II kinases and inhibitors of these kinases and PKC reversed chemerin-stimulated MSC migration. Chemerin stimulation of MSCs also induced expression and secretion of macrophage inhibitory factor (MIF) that tended to restrict migratory responses to low concentrations of chemerin but not higher concentrations. In a xenograft model consisting of OE21 esophageal cancer cells and CAMs, homing of MSCs administered i.v. was inhibited by CCX832. Thus, chemerin secreted from esophageal cancer myofibroblasts is a potential chemoattractant for MSCs and its inhibition may delay tumor progression.

## Introduction

The importance of the tumor microenvironment in determining cancer cell growth and spread is now well recognised [1]. Stromal cell types that contribute to the microenvironment include inflammatory and immune cells, endothelial cells, pericytes and fibroblast cell lineages [2]. In the case of the latter a growing body of evidence indicates that cancer-associated fibroblasts (CAFs), of which myofibroblasts are a prominent subtype, differ from their counterparts in normal tissue [3], [4], [5]. There is also a growing appreciation that bone marrow derived mesenchymal stromal (stem) cells (MSCs) can influence cancer progression by migration to tumor sites where they may differentiate into a variety of cell types including myofibroblasts [6], [7]; they may also be useful as vehicles to provide targeted anticancer therapy [8]. Although there is evidence for chemokine involvement in MSC recruitment the mechanisms remain poorly understood [9], [10].

Esophageal cancer is considered to account for nearly half a million deaths a year worldwide. Adenocarcinoma, associated with reflux and obesity, arises on a background of Barrett's esophagus and is increasing in incidence in Western societies; esophageal squamous cell carcinoma (ESCC) is associated with smoking, alcohol intake and poor diet and is of high incidence in developing countries [11]. There is a growing appreciation of the role of CAFs/myofibroblasts in ESCC particularly in promoting cancer invasion and angiogenesis although in general these remain poorly understood [12], [13].

Chemerin (tazarotene induced gene 2, TIG2; retinoic acid receptor responder 2, RARRES2) is an 18 kDa chemokine-like protein that acts at ChemR23 (chemokine-like receptor 1, CMKLR1) [14], [15]. It is secreted as an inactive precursor that is activated by a variety of extracellular proteases which remove a C-terminal hexapeptide to liberate a 157 amino acid active form; it is expressed in adipocytes, liver and placenta and has roles in adipogenesis and leukocyte chemotaxis including the recruitment of dendritic and natural killer (NK) cells to sites of inflammation or cancer [16], [17], [18], [19]. In the present study we identified increased expression of chemerin in ESCC cancer-associated myofibrobroblasts (CAMs) compared with adjacent tissue myofibroblasts (ATMs), and found expression of its cognate receptor ChemR23 by MSCs. We therefore hypothesised that chemerin acts as an MSC chemoattractant and we present here evidence to support the hypothesis.

## Materials and Methods

### Cells

Myofibroblasts were generated from tumors and adjacent tissue of patients with ESCC using previously described methods (Table S1 in File S1) [20], [21], and were used between passages 3 and 10. This work was approved by the Ethics Committee of the University of Szeged, Hungary and all subjects gave informed consent. ESCC cells (OE21) and human umbilical vein endothelial cells (HUVEC) were obtained from American Type Culture Collection (Manassas, VA). Human bone marrow derived mesenchymal stem cells were used at passages 3-12 in their undifferentiated state; up to passage 12 they exhibited adipocyte, osteocyte and chondrocyte differentiation in adipocyte, osteocyte and chondrocyte differentiation media (Lonza, Cambridge, UK); the cells were CD105, CD166, CD29, CD44, α-SMA and vimentin positive and were CD14, CD34, CD45, cytokeratin and desmin negative.

### Cell Culture

Myofibroblasts were cultured as previously described [20]. MSCs were maintained in an undifferentiated state in MSCGM (Lonza) containing basal medium and MSC growth supplements. Cells were maintained at 37°C in 5% v/v CO_2_; HUVECs were maintained in EGM medium and were used at passages 5 to 9; OE21 cells were cultured in RPMI-1640 supplemented with 10% v/v FBS, 1% v/v penicillin-streptomycin, 2% v/v L-glutamine.

### Conditioned media

Myofibroblasts (1.5×10^6^ cells) were plated in T-75 falcon flasks and maintained at 37°C in 5% v/v CO_2_ for 24 h in full media (FM). Cultures were then washed 3 times with sterile PBS and incubated in 15 ml serum free (SF) media for 24 h. Conditioned media (CM) were collected, centrifuged (7 min, 800×g, 4°C) and aliquots were stored at −80°C until further use.

### Immunocytochemistry

Cells were paraformaldehyde(PFA)-fixed (4% w/v), permeabilised with 0.2% Triton X-100 in PBS (PBS-T) for 30 min and processed for immunocytochemistry as previously described [22] using primary antibodies to CD105, CD34, CD29 (Ancell Corporation, Bayport, MN), α-SMA, vimentin, desmin, pancytokeratin (Fitzgerald, NJ), ChemR23 (Millipore, MA,) or GPR1 (Abcam, Cambridge, UK) followed by incubation with the appropriate fluorescein or Texas Red labelled secondary antibodies raised in donkey (Jackson Immunoresearch, Soham, UK), and mounted with Vectashield^®^ containing DAPI (Vector Laboratories, Peterborough, UK). Slides were viewed using a Zeiss Axioplan-2 microscope (Zeiss Vision, Welwyn Garden City, UK). Images were captured using a JVC-3 charge-coupled device camera at 40× magnification with KS300 software (Imaging Associates, Bicester, Oxfordshire, UK).

### Proteomic analysis

The secretome of esophageal myofibroblasts was studied after iTRAQ labelling of proteins in media followed by LC-MS/MS as previously described (see Methods S1) [3]. Putative chemerin targets in MSCs were sought after SILAC labelling of cells followed by exposure to chemerin (R&D Systems, Abindgon, Oxfordshire, UK) for 24 h and processing of cells for LC-MS/MS (see Methods S1) as previously described [23].

### Western blotting

Media or cell extracts prepared in RIPA buffer containing protease and phosphatase inhibitors were resolved by SDS-PAGE electrophoresis and processed for Western blotting as previously described [24] using antibodies to chemerin, macrophage migration inhibiting factor (MIF), matrix metalloproteinase (MMP)-2 (R&D Systems), GAPDH (Biodesign, Maine), total and phosphorylated p42/44, p38 and JNK-II kinases (Cell Signaling, Massachusetts).

### ELISA

ELISAs for chemerin (Adipo Bioscience, CA, USA) and MIF (R&D Systems) were applied to myofibroblast media according to the manufacturer's instructions.

### Cell migration assays

Transwell migration assays were performed using BD inserts (BD Bioscience, California) as previous described [25] employing chemerin (R&D Systems), chemerin-9 (Piscataway, NJ) or undiluted CM in the lower well. The effects were studied of phorbol 12-myristate 13-acetate (PMA), Ro320432, SB202190, SP600125, U0126, ISO-1 (Calbiochem, Darmstadt, Germany), LY294002 (New England labs, Hertfordshire, UK), chemerin neutralising antibody (MAb2325, R&D Systems), CCX-832 and CCX826 (ChemoCentryx, Mountain View, CA) [26]. Scratch wound migration assays were performed as previously described [27]. Transendothelial migration assays were performed using MSCs labelled with 1 µM PKH67 (Sigma Aldrich, Dorset, UK) according to the manufacturer's instructions and BD BioCoat Matrigel Invasion Chambers (BD Bioscience) coated with a monolayer of HUVECs 48 h previously. Migrating MSCs were subsequently counted as fluorescent cells.

### MMP-2 activity assays

MMP-2 activity in MSC media was determined by a selective fluorescence substrate, MCA-Pro-Leu-Ala-Nva-Dpa-Ala-Arg-NH_2_, according the manufacturer's instructions (Merk Biosciences, Beeston, Nottingham, UK).

### Chemerin, chemR23, GPR-1 and MIF knockdown

Myofibroblasts were transfected with 3 different silencing RNAs (siRNA, Table S2 in File S1)(3 µM) for chemerin (Sigma, UK). MSCs were treated with three different siRNAs (Table S2 in File S1)(3 µM) for chemR23, and validated siRNAs for GPR-1 and MIF (Invitrogen, Paisley, UK). The efficiency of knockdown was verified by Western blotting or immunocytochemistry.

### Transient transfection

Cells were transfected using Amaxa Fibroblasts Nucleofector kits (Amaxa; Köln, Germany) and AmaxaTM Human MSC Nucleofector kits (Amaxa; Köln, Germany) according to manufacturer's instructions. Each transfection employed 2 µg of DNA (5×10^5^ cells) and 100 µl of complete nucleofector solution. Transfection was achieved using program U-23 (for high transfection efficiency) by adding 500 µl of the pre-warmed culture medium to the cuvette and transfer of samples to T-75 flasks with 20 ml of freshly prepared medium.

### MSC homing to xenografts

All animal experiments were conducted after approval by the University of Liverpool Animal Welfare Committee and were in compliance with the U.K. Animals (Scientific Procedures) Act 1986 (PPL 40/3137).To study MSC recruitment to xenografts, 6-8 week-old immunocompromised BALB/c nu/nu mice (Charles River, Wilmington, MA) were injected s.c. with OE21 cells (10^6^) alone or with CAMs (5×10^5^). Mice with tumors of comparable size (1.0–1.2 cm diameter) subsequently received CCX832 (2 mg/ml, 125 µl, i.v.), or vehicle, followed after 24 h by a second dose and MSCs labelled with PKH67 (7.5×10^5^). After 24 h, tumours were dissected, fixed in 4% PFA and processed for localisation of fluorescent cells.

### Statistics

The final results were calculated as mean ± standard error of means (SEM). Student t-test and ANOVA were performed on the data as appropriate with significance at p≤0.05 using Systat Software Inc. (London, UK) unless otherwise stated.

## Results

### Increased chemerin expression in squamous esophageal CAMS

**Figure 1:**
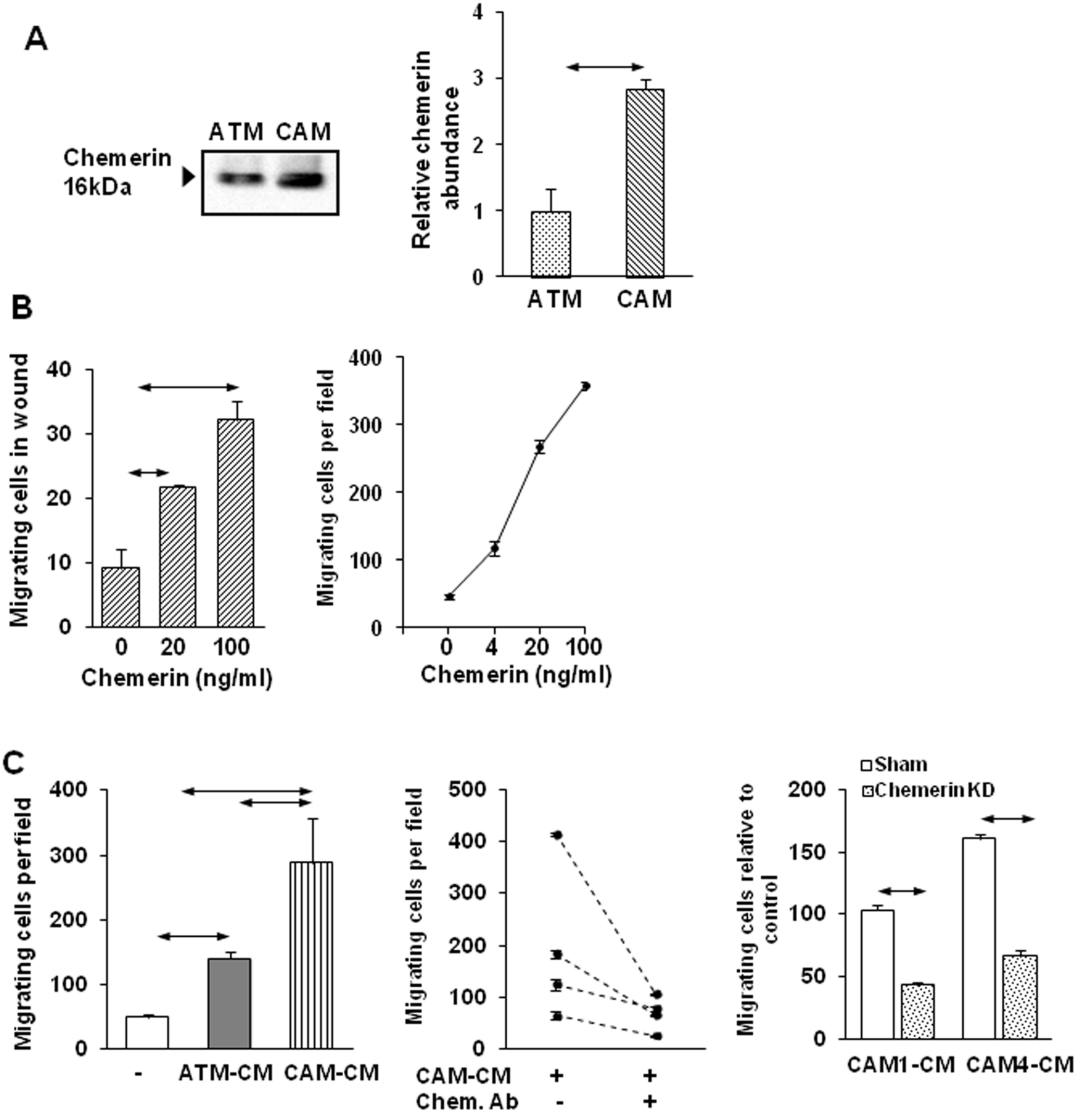
Chemerin exhibits increased expression in CAMs and stimulates MSC migration. *A*. Representative Western analysis of chemerin in media from ESCC CAMs and ATMs (left). Quantitative analysis by densitometry of chemerin abundance in media from ESCC CAMs and ATMs (n = 4 different pairs of myofibroblasts) (right). *B*. Concentration-dependent stimulation of MSC migration by chemerin in scratch wound migration assays (left) and Boyden chamber migration assays (right)(n = 3). *C*. Increased migration of MSCs in Boyden chambers in response to conditioned media (CM) from CAMs and their respective ATMs (left) (n = 4 different pairs of myofibroblasts). Stimulation of MSC migration by CAM-CM was inhibited by chemerin neutralizing antibody (Chem.Ab; 10 µg/ml) (center). MSC migration was decreased in response to CM from CAM1 and CAM4 cells transfected with chemerin siRNA#3 (right). Horizontal arrows, p<0.05, t- test (n = 3).

Myofibroblasts identified by α-SMA expression were found in greater numbers and exhibited disrupted morphology and architecture in ESCC compared with adjacent tissue (Fig S1 in File S2). Cultured myofibroblasts from these tumors and adjacent tissue expressed α-SMA and vimentin, but not desmin or cytokeratin; CM from CAMs stimulated growth and migration of OE21 cells compared with ATM-CM (Figs S2, S3 in File S2). Using iTRAQ labeling followed by LC-MS/MS to identify potential cell signalling molecules secreted by CAMs, we found increased chemerin abundance in CAM media compared with ATM from all of four pairs of ESCC samples (Fig S4 in File S2; Table S3 in File S1). Western blotting confirmed increased chemerin in CAM compared with ATM media (Fig 1A), and ELISA of media indicated 2.1±0.4 fold higher chemerin secretion by CAMs *vs* ATMs. In cell extracts, Western blots showed modest but significantly elevated chemerin in CAMs compared with their paired ATMs (Fig S5 in File S2). A putative chemerin receptor, ChemR23, was expressed by MSCs (see below), and chemerin stimulated concentration-dependent migration of MSCs in two different assays (Fig 1B). Moreover, CAM-CM stimulated MSC migration and the response was significantly greater than to ATM-CM (Fig 1C, left). Direct evidence for a role of chemerin as an active component of CAM-CM was provided by the observation that immunoneutralisation of chemerin inhibited the effect of CAM-CM (Fig 1C, center); additionally, siRNA knockdown of chemerin expression decreased the activity of CM subsequently applied to MSCs in migration assays (Fig 1C, right; Fig S6 in File S2).

### ChemR23 expressed by MSCs mediates migratory responses to chemerin

**Figure 2:**
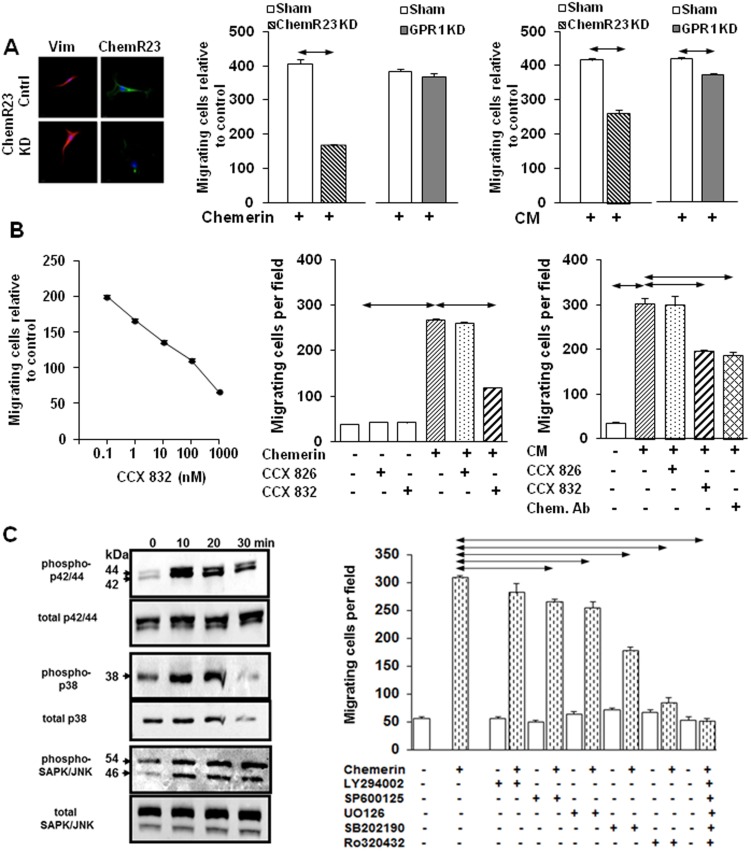
ChemR23 mediates chemerin stimulation of MSC migration via PKC and MAP kinases. *A*, Representative images from MSCs stained for vimentin (positive control) and chemR23 revealing knock-down (KD) after ChemR23 siRNA treatment (left). Knockdown of ChemR23, but not GPR1, inhibited MSC migration in response to chemerin (100 ng/ml)(center) and CAM-CM (right). *B*, Concentration-dependent inhibition of MSC migration in response to chemerin by the ChemR23 antagonist CCX832 (left) but not the control compound CCX826 (1 µM) (center). MSC migration in response to CAM-CM was inhibited similarly by chemerin neutralising antibody, and CCX832, but not CCX826 (1 µM)(right). *C*, Representative Western blot shows increased phosphorylation of p42/44, p38 and JNK-II kinases in MSCs treated with chemerin (100 ng/ml)(left). In Boyden chamber assays, chemerin-stimulated MSC migration was inhibited by the JNK-II inhibitor, SP600125 (50 µM), the p42/44 inhibitor, UO126 (10 µM), p38 inhibitor SB202190 (3 µM), and the PKC inhibitor Ro320432 (2 µM) but not by PIK3 inhibitor LY294002 (50 µM) (right). Horizontal arrows, p<0.05, ANOVA (n = 3 in each case).

The expression in MSCs of the putative chemerin receptor, ChemR23, has been identified by gene array [28] and we confirmed expression of this and a second putative receptor, GPR1 [29], by immunocytochemistry. Knockdown by siRNA of ChemR23 (Fig 2A, left; Fig S7 in File S2) significantly inhibited the migratory response to both chemerin (Fig 2A, center) and CAM-CM (Fig 2A, right), while knockdown of GPR1 had little effect. A ChemR23 antagonist, CCX832, dose-dependently inhibited MSC migration in response to chemerin (Fig 2B, left and center) and CAM-CM (Fig 2B, right) while an inactive analogue, CCX826 had no effect; the inhibition of CAM-CM by CCX832 was similar to that achieved by immunoneutralisation (Fig 2B, right).

### Chemerin stimulation of protein kinase pathways mediates MSC migratory responses

Chemerin promptly increased phosphorylation of several protein kinases including p42/44, p38 and JnkII kinases in MSCs (Fig 2C, left). Inhibitors of all three kinases significantly reduced the migratory response of MSCs to chemerin but inhibition of PI-3-kinase using LY294002 had no effect. The PKC inhibitor Ro320432 also inhibited migration, and the combination of Ro320432, U0126, SP600125 and SB202190 completely inhibited migratory responses (Fig 2C, right). Evidence that PKC activation was upstream of MAP kinase stimulation is provided by the observation that PMA stimulated MSC migration and this was inhibited by U0126, SP600125 and SB202190; moreover, PMA stimulated phosphorylation of p42/44, p38 and JnkII kinases (Fig S8 in File S2). There was a marked reduction in phosphorylation of p42/44, p38 and JnkII kinases after ChemR23 knockdown using siRNA consistent with the idea that these kinases are downstream of ChemR23 (Fig S8 in File S2).

### Chemerin increases MIF expression in MSCs which restrains migration

**Figure 3:**
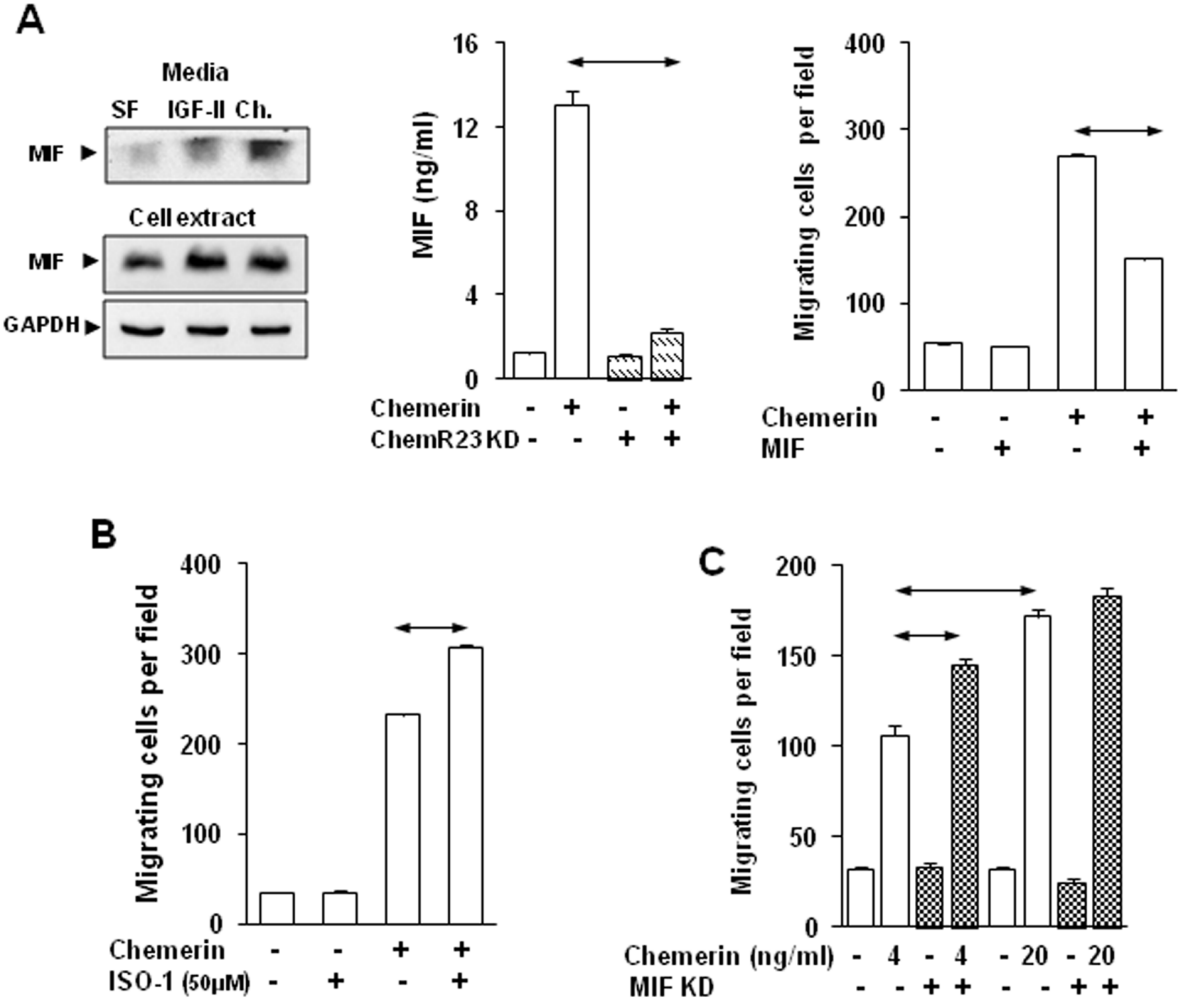
Chemerin increased MIF secretion by MSCs which restrains migratory responses. *A*, Representative Western blots showing MIF in MSC media (top left) and cell extracts (bottom left) treated with chemerin (Ch; 100 ng/ml) or IGF-II (100 ng/ml) for 15 min. ChemR23 knock-down decreased MIF release in response to chemerin (center). Chemerin-stimulated MSC migration was inhibited by MIF (200 ng/ml)(right). *B*, Suppression of MIF signaling with ISO-I (50 µM) further increased chemerin-stimulated migration. *C*, MIF knock-down in MSCs increased migration in response to 4 ng/ml, but not 20 ng/ml chemerin. Horizontal arrows, p<0.05, t- test (n = 3).

In order to further define putative targets of chemerin we applied SILAC and LC-MS/MS to the identification of proteins in cell extracts and media of MSCs treated with chemerin for 24 h. Unexpectedly, MIF was increased in chemerin-stimulated cells (Table S4 in File S1; Fig S9 in File S2). Western blot verified that chemerin, as well as IGF-II used as a positive control, increased MIF in cell extracts and media (Fig 3A, left), and using ELISA there was approximately 10-fold higher MIF concentrations in media after chemerin treatment. Following ChemR23 knockdown the MIF response to chemerin was profoundly inhibited (Fig 3A, center). In the presence of MIF, the MSC migratory response to chemerin was inhibited by approximately 50% (Fig 3A, right). To determine the functional significance of MIF in MSC migration we employed the MIF antagonist ISO-1; this suppressed the effect of MIF in inhibiting chemerin-stimulated MSC migration, and significantly increased the migratory response of MSCs to chemerin (Fig 3B). Similarly, MIF knockdown (Fig S10 in File S2) increased MSC migration in response to low concentrations of chemerin (4 ng/ml), but interestingly the response to chemerin at higher concentrations (20 ng/ml) was not influenced by MIF knockdown (Fig 3C).

### Chemerin stimulates MSC migration across endothelial cells and requires MMP-2

**Figure 4:**
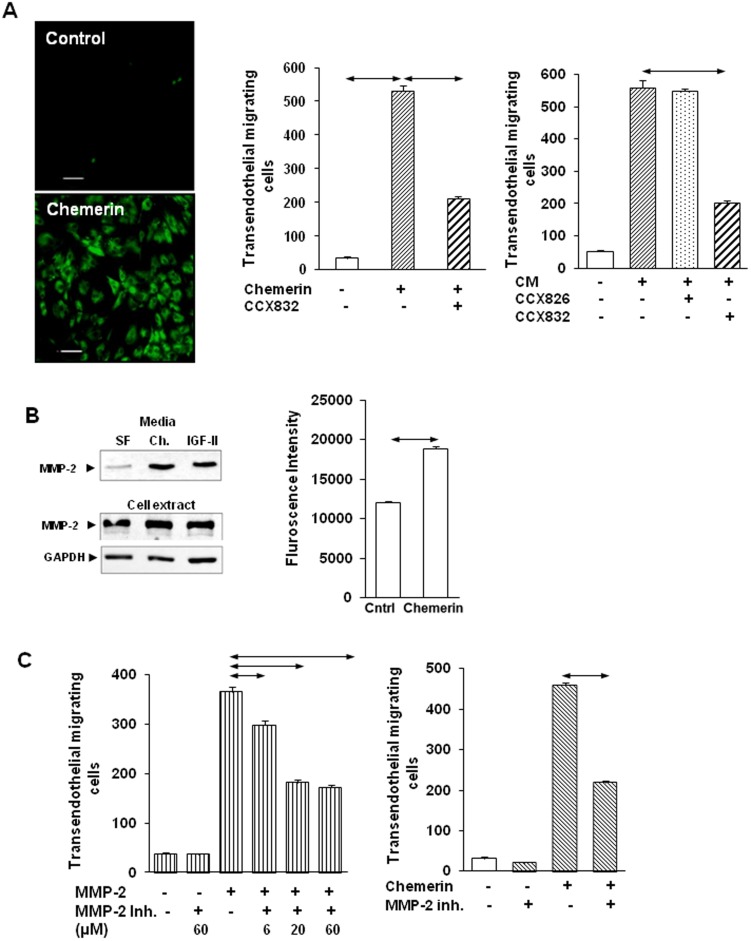
Chemerin stimulates transendothelial migration of MSCs and requires MMP-2. *A*, Representative fields from MSC transendothelial migration experiments showing migration of PKH67-labelled MSCs (left). CCX832 (1 µM) inhibited chemerin- (center) and CAM-CM stimulated MSC transendothelial migration but CCX826 (1 µM) had no effect (right). *B*, Chemerin, and IGF-II used as a positive control, promptly (30 min) stimulated proMMP2 abundance in media as detected by Western blot but had no effect on cellular proMMP2 abundance (left); chemerin significantly increased MMP-2 enzyme activity in MSC media detected by the selective substrate MCA-Pro-Leu-Ala-Nva-Dpa-Ala-Arg-NH_2_ (right). *C*, Human recombinant MMP-2 (80 ng/ml) stimulated transendothelial migration and there was dose-dependent inhibition by an MMP-2 selective inhibitor (MMP-2 inhibitor I) (left). The MMP-2 inhibitor (60 µM) significantly inhibited chemerin-stimulated MSC transendothelial migration (centre). Horizontal arrows, p<0.05, t- test (n = 3).

The data implicate chemerin in the recruitment of MSCs to CAM-containing cancer microenvironments. *In vivo* this requires transendothelial migration and so we examined whether chemerin was able to stimulate MSC migration through a monolayer of endothelial cells previously formed on Boyden chambers. MSCs labelled with PKH67 (Fig 4A, left) were identified as migrating in response to chemerin (Fig 4A, center) and CAM-CM (Fig 4A, right), and the migratory response was inhibited by CCX832 but not CCX826. Secreted proteases that might facilitate transendothelial migration were then sought in the protein lists obtained from SILAC-labelled MSCs treated with chemerin. Abundant MMP-2 was identified in chemerin-stimulated samples (Table S5 in File S1) and Western blots (Fig 4B, left) and MMP-2 enzyme activity assays (Fig 4B, right) confirmed increased abundance in media in response to chemerin. In migration studies, addition of MMP-2 stimulated MSC migration and this was significantly reduced by a MMP-2 inhibitor (Fig 4C left). The same inhibitor also significantly reduced MSC transendothelial migration in response to chemerin (Fig 4C, right).

### In a xenograft model, MSC homing is stimulated by CAMs and inhibited by CCX832

**Figure 5:**
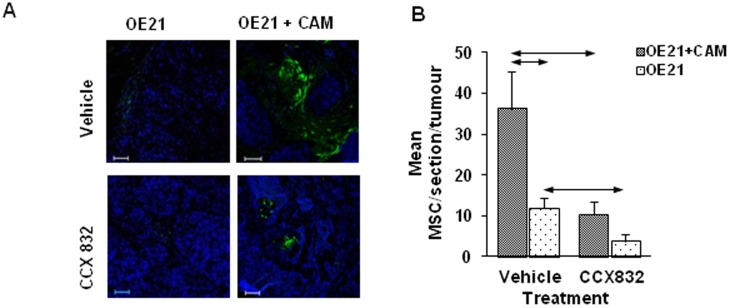
Increased MSC homing to xenografts seeded with CAMs and inhibition of homing by the chemR23 receptor antagonist, CCX832. *A*, Visualisation of PKH67-labelled MSCs in representative fields from xenografts established with OE21 cancer cells alone or co-injected with CAMs followed by treatment with vehicle (top) or CCX832 (bottom) and iv injection of PKH67-labelled MSCs. *B*, In xenografts with OE21 cancer cells and CAMs there was increased MSC homing expressed as labelled cells per unit area of xenograft compared with xenografts of OE21 cancer cell alone; treatment with CCX832 inhibited homing (OE21/vehicle, n = 3; OE21/CCX832, n = 4; OE21 and CAMs/vehicle, n = 6; OE21 and CAMs/CCX832, n = 6). Horizontal arrows, p<0.05, ANOVA.

On the basis of the data described above, we hypothesised that *in vivo* chemerin mediates MSC homing to tumors consisting of cancer cells and CAMs. In a xenograft model of OE21 cells in nude mice, matched tumors of similar size (1.0–1.2 cm diameter) generated with and without co-administration of CAMs were studied after subsequent i.v. injection of PKH67-labelled MSCs. Labelled cells in the xenografts were increased in tumors of OE21 and CAMs compared with OE21 cells alone (Fig 5A). Pretreatment of mice with the ChemR23 antagonist CCX832 prior to injecting MSCs significantly reduced the number of labelled cells in the xenografts thereby demonstrating a role for chemerin in MSC recruitment *in vivo* (Fig 5B).

## Discussion

There is increasing evidence that MSCs migrate to tumors where they contribute to tumor growth [6], [30]. The mechanisms of homing and migration remain incompletely understood. In this study we provide evidence that expression of the chemokine-like peptide, chemerin, is increased in CAMs from ESCC and acts as a chemoattractant for MSCs via activation of the G-protein coupled receptor ChemR23. A small molecule antagonist of ChemR23, CCX832, inhibited the action of chemerin both *in vitro* and in a xenograft model *in vivo*. The findings identify chemerin as a novel CAM-derived determinant of MSC recruitment to tumors.

Previous studies have reported a number of different roles for CAFs, CAMs and related cells in the progression and response to therapy of a variety of cancers including prostate, breast, stomach, lung and colon [2], [31], [32], [33], [34], [35]. The CAMs used for the present studies were derived from tumors in which myofibroblast number, architecture and morphology were disrupted; moreover CM from these CAMs evoked a more aggressive phenotype in cancer cells (proliferation, migration) compared with CM from ATMs. More generally, the actions of myofibroblasts are reported to be both positive and negative on cancer cell proliferation and migration, and there are also effects on angiogenesis, and modulation of immune mechanisms [3], [12]. However, while MSC homing to cancer sites is recognised, the specific role of myofibroblasts in this process has been unclear. The present data suggest not just that CAMs are active participants in MSC recruitment but also that one specific mediator, chemerin, is differentially expressed in CAMs and ATMs.

Chemerin emerged through a proteomic study of myofibroblast secretomes. There is growing interest in the application of proteomic studies to define the secretomes of stromal cells, but even so these remain less well studied than cancer cell secretomes. Previous secretome studies on both fibroblastic lineage cells and MSCs have identified some of the molecules found in the present study notably ECM proteins, MMPs, IGFBPs; signalling molecules identified in these studies include SDF-1, HGF, EGF and other chemokines [36], [37]. Our initial identification of chemerin was made in all four pairs of cells examined and was validated by Western blot and ELISA of media which together indicate that chemerin should be considered a novel mediator of myofibroblast cell signalling.

Chemerin is normally expressed by adipocytes, liver, lung and other cells. In a number of cancers, including squamous skin cancer, melanoma, prostate, lung, breast and hepatocellular carcinoma, decreased chemerin expression is associated with adverse outcome. However, it is worth noting that previous work has not, for the most part, taken account of different patterns of expression of chemerin in tumor epithelial cells compared with stromal cells [18], [38]. Since chemerin is a chemoattractant for NK cells and dendritic cells [14], [16], [19] it has been suggested that loss of chemerin allows tumors to evade the immune defense mechanisms that inhibit tumorigenesis [18], [38], [39]. However, esophageal cells may provide a tolerogenic environment [40] that mitigates the protective effects of chemerin. Moreover, in gastric cancer there is increased plasma chemerin and chemerin stimulates cancer cell invasion *in vitro* [41]. Thus, chemerin may exert both positive and negative effects on tumor progression.

There are multiple potential roles for chemerin released by CAMs including modulation of cancer and immune cell function and angiogenesis. We found that the cognate receptor ChemR23 was expressed by MSCs and that chemerin was a chemoattractant for these cells. Since receptor knockdown, immunoneutralisation and a ChemR23 receptor antagonist only partially inhibited the effects of CAM-CM, there are also likely to be other CAM chemoattractants. We suggest chemerin is a good candidate for further study not least because of the availability of receptor antagonists. The mechanisms of chemotaxis include activation of PKC and of multiple downstream kinase pathways including p42/44, p38 and JNK-II kinases, all of which seem to play a role. At least in part these intracellular signalling mechanisms may resemble those described in other cells that exhibit chemerin-stimulated chemotaxis including dendritic cells [14].

Previous studies have shown that a variety of growth factors and chemokines stimulate MSC migration including HGF, TNFα, SDF-1, CXCL12, CXCL13, CHCL16 and CCL22 [42], [43]. MSCs transmigrate across endothelia by chemokine-mediated mechanisms that also involve matrix metalloproteinases notably MMP-2 [43], [44], [45], [46], [47]. We found rapid (30 min) stimulation of proMMP-2 secretion by MSCs in response to chemerin and a role for MMP-2 in facilitating chemerin-stimulated transendothelial migration, presumably following activation by other extracellular proteases such as MMP-14 [45].

It is known that MIF inhibits MSC migration [48], [49]. The present data suggest that concentration-dependent induction of MIF in MSCs by chemerin acts to restrain migration. However, the inhibitory effect is attenuated at high concentrations of chemerin. One implication is that control myofibroblasts, where chemerin expression is modest, do not effectively promote MSC recruitment because of the autoinhibitory action of MIF; however in CAMs where there is increased chemerin, the capacity for MSC recruitment is enhanced since the autoinhibitory effect of MIF is overcome.

The ChemR23 antagonist CCX832 has previously been used to define novel interactions between perivascular adipocytes and vasoconstrictor responses [26]. We now show both *in vitro* and *in vivo* in a xenograft model that CCX832 inhibits MSC migration in response to CAMs. Whether chemerin influences MSC differentiation after recruitment to cancers remains to be determined. It would not be surprising if it did, since it is known to stimulate mesenchymal stem cell adipogenesis [17], [50]. Taken as a whole the present data indicate chemerin is a novel potential regulator of cancer progression by targeting MSC recruitment and suggest the feasibility of using ChemR23 receptor antagonists to regulate this process.

## Supporting Information

Methods S1 **Supplementary Methods.**
(PDF)

File S1 **Supplementary Tables.** Table S1 to Table S5.
(PDF)

File S2 **Supplementary Figures.** Figure S1 to Figure S10.
(PDF)
