# Supplementary material for: Increased Expression of Chemerin in Squamous Esophageal Cancer Myofibroblasts and Role in Recruitment of Mesenchymal Stromal Cells
Source: PLoS One. 2014 Aug 15;9(8):e104877. doi: 10.1371/journal.pone.0104877 (PMC4134237; doi:10.1371/journal.pone.0104877)
Supplement: Methods S1 — Supplementary Methods. (PDF) [file pone.0104877.s001.pdf]

Supplementary Methods

Isobaric tagging for relative and absolute quantitation (iTRAQ). Myofibroblast CM was concentrated to 500 µl, and 80 µg protein was precipitated in cold acetone, resuspended in 0.5M triethylammonium bicarbonate (TEAB) in 0.1% SDS. Samples were reduced with 2µl 50mM tris-(2-carboxyethyl) phosphine (TCEP) at 60°C for one hour and alkylated with 1µl 200mM methyl methanethiosulfonate (MMTS) for 10 min at room temperature Myofibroblasts were lysed directly in TEAB/SDS. Samples (100µg) were labelled using the 4-plex iTRAQ® kit (AB SCIEX, Foster City, CA, USA) according to manufacturer's instructions. Labelled samples were pre-fractionated using a PolyLC PolySULFOETHYL A (4.6 x 200mm i.d.) cation exchange (CEX) column using an Agilent 1100 HPLC system (Agilent Technologies, Santa Clara, CA, USA). Samples were then analysed using an LC Packings Ultimate nano-LC system run in-line with a QStar Pulsar i mass spectrometer (AB Sciex). Protein identification and quantification were performed using the ProteinPilot™ v3.0.1 software (AB Sciex). The Paragon algorithm was selected as the default search program, with the digestion agent set as trypsin and cysteine modification as methyl methanethiosulfonate. Proteins were reported based on the assignment of at least two tryptic peptides with a confidence >95%, or on the assignment of one tryptic peptide with a confidence >99% and a local FDR calculated using the PSPEP algorithm of <1%. Proteins exhibiting a differential abundance in CAMs *vs* ATMs were calculated only on the assignment of at least two tryptic peptides using a Pro Group™ algorithm of ProteinPilot™. When comparing groups of patients, proteins that were identified in >80% in one of the groups were used in the analysis.

SILAC labelling. For SILAC experiments, cells were cultured in media supplemented with either $^{12}C_6$ lysine and $^{12}C_6$ arginine (Light label), or $^{13}C_6$ lysine and $^{13}C_6$ arginine (Heavy label) for at least 6 population doubling times. The Human Mesenchymal Stem Cell SILAC kit from Pierce was used and the media supplemented with 1% (v/v) penicillin/streptomycin (Sigma) and 1% (v/v) L-glutamine (Sigma). Untreated hMSCs were grown in heavy media, whilst hMSCs treated with chemerin (R&D, 100ng/mL) for 24 h were grown in light media. hMSCs were plated at 1 x10^6 cells per 10cm diameter dish and grown for 24h in full media. Cells were then washed three times with PBS, and cultured in serum-free medium with or without chemerin. Medium was collected after 24h and centrifuged at 800 x g for 7 min to remove debris. Cells were scraped in 100µL RIPA buffer, lysed on ice, sonicated and clarified by centrifugation before storing at -80°C.

StrataClean resin (Agilent) was used to capture proteins in media samples. Strataclean beads (10µL per mL),were mixed for 1 min and then separated by centrifugation. The beads were washed three times with 25mM ammonium bicarbonate and StrataClean-bound protein samples were resuspended in 25mM ammonium bicarbonate, denatured by addition of 0.05% (w/v) RapiGest (Waters) and incubated at 80°C for 10 min. Samples were then reduced by 3mM DTT at 60°C for 10min, and alkylated by 9mM iodoacetamide at room temperature for 30 min. Samples were then digested by addition of sequencing-grade trypsin in a roughly 50:1 protein:trypsin ratio and incubated at 37°C for 18 h.

Cell extracts in RIPA buffer were assayed using the DC Protein Assay (BioRad). Samples were reduced by addition of DTT to a final concentration of 100mM and processed using the method described by Wiśniewski et al (1). Briefly, up to 100µg of protein was loaded onto Amicon Ultra 10K centrifugal filters (Millipore), together with 200µL buffer UA (8M urea in 0.1M Tris/HCl, pH8.5) and centrifuged at 14,000 x **g** for 40 min, followed by a wash with a further 200µL buffer UA. Proteins were alkylated by addition of iodoacetamide to a final concentration of 50mM in buffer UA. Samples were mixed for 1min, incubated at room temperature for 5minutes, and centrifuged for 30 min. This was followed by two washes of 100µL buffer UA, then two washes of 100µL 50mM ammonium bicarbonate. Proteins were then digested by addition of 40µL 0.05µg/µL sequencing grade trypsin in ammonium bicarbonate, and incubated for 18 h at 37°C. Digested peptides were collected in a fresh collection tube by centrifugation at 14,000 x g for 10 min, followed by a further elution wash with 50µL 0.5M NaCl. Samples were then desalted using C18 ZipTips (Millipore), dried, and resuspended in 20µL 3% acetonitrile, 0.1% formic acid.

Samples were analysed on a Nano-Acquity (Waters) reverse phase HPLC system in-line with an LTQ Orbitrap Velos (Thermo). SILAC data were searched and analysed using MaxQuant 1.1.1.36 (2) against the human IPI database v3.68 using the recommended default settings. Oxidation (M) and acetylation (protein N-term) were set as variable modifications, and carbamidomethylation (C) was set as a fixed modification. The first search function was enabled, MS/MS tolerance set to 0.5Da and the number of top peaks per 100Da set to 6. The data were also searched against a database of known contaminants. For identification, both the peptide and protein FDRs were set to 1%. For quantification, the 'filter labelled amino acids' box was unchecked, both razor and unique peptides selected for quantification and the minimum ratio count for quantification set to 1.

References

1. Wisniewski JR, Zougman A, Nagaraj N, Mann M. Universal sample preparation method for proteome analysis. Nature methods. 2009;6:359-62.

2. Cox J, Mann M. MaxQuant enables high peptide identification rates, individualized p.p.b.-range mass accuracies and proteome-wide protein quantification. Nature biotechnology. 2008;26:1367-72.
