# Supplementary material for: Increased Expression of Chemerin in Squamous Esophageal Cancer Myofibroblasts and Role in Recruitment of Mesenchymal Stromal Cells
Source: PLoS One. 2014 Aug 15;9(8):e104877. doi: 10.1371/journal.pone.0104877 (PMC4134237; doi:10.1371/journal.pone.0104877)
Supplement: File S1 — Supplementary Tables. Table S1 to Table S5. (PDF) [file pone.0104877.s002.pdf]

SUPPLEMENTARY TABLE S1

Patient #	Age	Gender	Survival (months)	Tumor Staging	Tumor Differentiation	Tumor Location	Classification	Adjacent Tissue
1	56	M	34	pT3N1M0	G3	esophagus	squamous carcinoma	normal
2	52	M	31	pT3N1M0	G2-3	esophagus	squamous carcinoma	normal
3	49	M	35	pT2N1M0	G2	esophagus	squamous carcinoma	GERD
4	69	M	14	pT3N1M0	G2	esophagus	squamous carcinoma	normal

Supplementary Table S1. Characteristics of patients used to provide esophageal myofibroblasts. Age, gender, post-operative survival, tumor staging, tumor localisation, tumor differentiation, and pathology assessment of tissue taken adjacent to the tumor, for patients used to provide CAMs and ATMs (see also Supplementary Figure 1, for information on myofibroblasts in these tumors).

SUPPLEMENTARY TABLE S2

Target	Sense	Antisense
Chemerin #1	GGGCUCUGAGGACAAAGUU[dT][dT]	AACUUUGUCCUCAGAGCCC[dT][dT]
Chemerin #2	CGCCCUUCCCAGCUGGAAU[dT][dT]	AUUCCAGCUGGGAAGGGCG[dT][dT]
Chemerin #3	GAAAUGCCUGGCCUGCAUC[dT][dT]	GAUGCAGGCCAGGCAUUUC[dT][dT]
ChemR23 #1	CUCUUCUCUCGCCUGGUCA[dT][dT]	UGACCAGGCGAGAGAAGAG[dT][dT]
ChemR23 #2	CAGUGAACAUGGUCUGGUU[dT][dT]	AACCAGACCAUGUUCACUG[dT][dT]
ChemR23 #3	CCAUGUGCAAGAUCAGCAA[dT][dT]	UUGCUGAUCUUGCACAUGG[dT][dT]
MIF	CGGACAGGGUCUACAUCAAtt	UUGAUGUAGACCCUGUCCGgg
GPR1	GCCUUAAGUCUUAAUAUUATT	UAAUAUUAAGACUUAAGGCTT

Supplementary Table S2. Sequences (5’>3’) of sense and antisense strands used for siRNA knockdown of chemerin, ChemR23, MIF and GPR1.

SUPPLEMENTARY TABLE S3

Name	Uniprot	Localisation	ID	Ratio CAMs/ATMs 1	2	3	4
Brain acid soluble protein 1	P80723	Membrane	BASP1_HUMAN			-1.25	1.42
CD44 antigen OS=Homo sapiens	P16070	Membrane	CD44_HUMAN			-1.31	-1.06
Calsyntenin-1	O94985	Membrane	CSTN1_HUMAN			-1.09	1.17
Transmembrane glycoprotein NM	Q14956	Membrane	GPNMB_HUMAN			-1.08	-1.22
Lactadherin OS=Homo sapiens (	Q08431	Membrane	MFGM_HUMAN			-1.39	1.68
Protein disulfide-isomerase	P07237	Membrane	PDIA1_HUMAN	-1.03	-1.04	1.25	1.18
Thy-1 membrane glycoprotein	P04216	Membrane	THY1_HUMAN		-1.07	-2.08	2.18
Vasorin OS=Homo sapiens GN=\	Q6EMK4	Membrane/Secreted	VASN_HUMAN			-1.06	1.84
Alpha-2-macroglobulin	P01023	Secreted	A2MG_HUMAN	-1.07	-1.34	1.30	-2.99
Annexin A1	P04083	Secreted	ANXA1_HUMAN			-1.44	-1.07
Annexin A2	P07355	Secreted	ANXA2_HUMAN	1.03	1.20		2.05
Annexin A5	P08758	Secreted	ANXA5_HUMAN	1.14	-1.24	-2.10	1.34
Beta-2-microglobulin	P61769	Secreted	B2MG_HUMAN	1.37	2.17	1.57	1.28
Transforming growth factor-beta-	Q15582	Secreted	BGH3_HUMAN	1.45	-1.08	-1.17	2.03
Complement C1r subcomponent	P00736	Secreted	C1R_HUMAN	1.14	1.53	2.12	1.67
Complement C1s subcomponent	P09871	Secreted	C1S_HUMAN	1.49	-1.00	1.56	2.12
45 kDa calcium-binding protein	Q9BRK5	Secreted	CAB45_HUMAN	1.94	1.68	1.48	1.94
Calreticulin	P27797	Secreted	CALR_HUMAN	-1.06	1.46	-1.80	-1.61
Calumenin	O43852	Secreted	CALU_HUMAN	1.40	-1.40	1.35	
Cathepsin B	P07858	Secreted	CATB_HUMAN	1.35	1.41	-1.22	2.33
Cathepsin D	P07339	Secreted	CATD_HUMAN			-1.75	1.72
Cathepsin L1	P07711	Secreted	CATL1_HUMAN	1.66	1.68		1.25
Coiled-coil domain-containing pro	Q76M96	Secreted	CCD80_HUMAN	-2.01	2.06		
Chitinase-3-like protein 1	P36222	Secreted	CH3L1_HUMAN	-1.30	6.59	6.43	7.74
Clusterin	P10909	Secreted	CLUS_HUMAN	1.57	-1.13	2.23	2.14
Collagen alpha-1(I) chain	P02452	Secreted	CO1A1_HUMAN	1.20	4.53	5.87	4.35
Collagen alpha-2(I) chain	P08123	Secreted	CO1A2_HUMAN	-1.22	1.87	7.16	3.74
Collagen alpha-1(III) chain	P02461	Secreted	CO3A1_HUMAN	-1.30	4.12	4.83	5.03
Collagen alpha-1(VI) chain	P12109	Secreted	CO6A1_HUMAN	1.76	1.00	1.21	1.17
Collagen alpha-2(VI) chain	P12110	Secreted	CO6A2_HUMAN	1.37	1.36	1.72	1.83
Collagen alpha-3(VI) chain	P12111	Secreted	CO6A3_HUMAN	-1.31	1.61	1.70	1.76

Collagen alpha-1(XII) chain	Q99715	Secreted	COCA1_HUMAN	1.43	1.78		
Versican core protein	P13611	Secreted	CSPG2_HUMAN	-1.82	2.77	1.63	-1.04
Protein CutA	O60888	Secreted	CUTA_HUMAN	-1.98	1.18		
Cystatin-C OS=Homo sapiens GI	P01034	Secreted	CYTC_HUMAN			2.02	1.34
Dickkopf-related protein 3	Q9UBP4	Secreted	DKK3_HUMAN	-1.02		1.39	2.44
Dipeptidyl peptidase 4	P27487	Secreted	DPP4_HUMAN	-1.32	-1.02		-1.10
Extracellular matrix protein 1	Q16610	Secreted	ECM1_HUMAN	-1.25	2.57	1.42	
Fibulin-1	P23142	Secreted	FBLN1_HUMAN	1.47	-1.20	1.64	
Fibulin-5	Q9UBX5	Secreted	FBLN5_HUMAN	1.75	-2.77	1.19	-1.02
Alpha-2-HS-glycoprotein	P02765	Secreted	FETUA_HUMAN	-1.17	-1.36	1.27	-2.22
Fibronectin	P02751	Secreted	FINC_HUMAN	1.03	2.25	2.33	2.60
Follistatin-related protein 1	Q12841	Secreted	FSTL1_HUMAN	1.12	1.74	3.78	1.28
Glia-derived nexin	P07093	Secreted	GDN_HUMAN	-2.63	-2.79	3.42	-4.69
Gelsolin	P06396	Secreted	GELS_HUMAN	-1.20	2.20	-1.20	1.83
Growth-regulated alpha protein	P09341	Secreted	GROA_HUMAN			-1.06	1.16
Serine protease HTRA1	Q92743	Secreted	HTRA1_HUMAN	1.14	2.24	1.32	2.09
Insulin-like growth factor-binding	P17936	Secreted	IBP3_HUMAN	-1.45	-1.73	1.49	
Insulin-like growth factor-binding	P22692	Secreted	IBP4_HUMAN	1.37	-1.08	2.31	1.24
Insulin-like growth factor-binding	P24593	Secreted	IBP5_HUMAN	-1.23	1.80	1.89	3.63
Insulin-like growth factor-binding	Q16270	Secreted	IBP7_HUMAN	1.30	1.72	1.30	1.84
Interleukin-8	P10145	Secreted	IL8_HUMAN			1.09	-1.73
Laminin subunit beta-1 OS=Hom	P07942	Secreted	LAMB1_HUMAN			2.00	1.07
Laminin subunit gamma-1	P11047	Secreted	LAMC1_HUMAN	1.41	1.11	1.97	1.65
Galectin-1	P09382	Secreted	LEG1_HUMAN	-1.07	1.66	-1.24	1.33
Galectin-3	P17931	Secreted	LEG3_HUMAN	-1.14	1.58	-1.03	1.57
Galectin-3-binding protein	Q08380	Secreted	LG3BP_HUMAN	1.22	1.16	1.60	1.16
Leukemia inhibitory factor	P15018	Secreted	LIF_HUMAN			-1.08	1.43
Latent-transforming growth facto	Q14767	Secreted	LTBP2_HUMAN			2.24	3.02
Lumican	P51884	Secreted	LUM_HUMAN	-2.10	2.05	4.74	1.58
Macrophage migration inhibitory f	P14174	Secreted	MIF_HUMAN	1.18	1.27	-1.33	-1.12
Interstitial collagenase	P03956	Secreted	MMP1_HUMAN	1.55	-2.52	-1.71	1.51
Stromelysin-2	P09238	Secreted	MMP10_HUMAN	1.02	-1.85	-3.46	2.55
72 kDa type IV collagenase	P08253	Secreted	MMP2_HUMAN	1.15	1.87	-1.27	1.17
Stromelysin-1	P08254	Secreted	MMP3_HUMAN	1.81	-3.08	-2.49	1.05

Epididymal secretory protein E1	P61916	Secreted	NPC2_HUMAN	1.08	-1.02	1.06	1.31
Plasminogen activator inhibitor 1	P05121	Secreted	PAI1_HUMAN	1.62	-2.69	2.85	-4.42
Pigment epithelium-derived facto	P36955	Secreted	PEDF_HUMAN	-1.05	1.45	2.75	1.41
Biglycan	P21810	Secreted	PGS1_HUMAN	1.00	2.24	3.77	
Decorin	P07585	Secreted	PGS2_HUMAN	-1.03	-1.16	1.93	1.21
Periostin	Q15063	Secreted	POSTN_HUMAN		1.22	3.22	
Sulfhydryl oxidase 1	O00391	Secreted	QSOX1_HUMAN	1.00	-1.02	1.41	1.29
Retinoic acid receptor responder	Q99969	Secreted	RARR2_HUMAN	1.66	4.63	1.83	4.70
Secreted frizzled-related protein	Q6FHJ7	Secreted	SFRP4_HUMAN	-1.46	6.19		
Spondin-2	Q9BUD6	Secreted	SPON2_HUMAN	-1.22	1.12	2.52	1.62
SPARC	P09486	Secreted	SPRC_HUMAN	1.01	3.21	4.67	2.07
Stanniocalcin-1	P52823	Secreted	STC1_HUMAN			-1.80	2.75
Stanniocalcin-2	O76061	Secreted	STC2_HUMAN	-1.67	-2.68		-2.81
Tissue factor pathway inhibitor 2	P48307	Secreted	TFPI2_HUMAN			-1.04	-1.63
Testican-1	Q08629	Secreted	TICN1_HUMAN	1.00	1.74	1.77	1.47
Metalloproteinase inhibitor 1	P01033	Secreted	TIMP1_HUMAN	-1.01	1.66	-1.18	1.05
Metalloproteinase inhibitor 2	P16035	Secreted	TIMP2_HUMAN	1.25	1.45	1.76	1.75
Lactotransferrin	P02788	Secreted	TRFL_HUMAN	-1.10	-2.30	1.56	-2.02
Thrombospondin-1	P07996	Secreted	TSP1_HUMAN	1.24	-1.51	2.60	-1.21
Thrombospondin-2	P35442	Secreted	TSP2_HUMAN	-1.09	2.08	1.71	1.95
Prostaglandin-H2 D-isomerase	P41222	Secreted/ER	PTGDS_HUMAN			4.44	5.08
Annexin A6	P08133	Secreted?	ANXA6_HUMAN			-3.08	2.28

Supplementary Table S3. Identification by iTRAQ of proteins in myofibroblast secretomes. Data are expressed as relative abundance in squamous cancer myofibroblasts relative to adjacent tissue myofibroblasts. Identifications limited to proteins found in at least two pairs of CAMs/ATMs. Note chemerin (highlighted) was increased in all four CAMs relative to their respective ATMs. The Table shows protein names, UnitProt IDs, status as a putative secretory peptide and data for four separate matched pairs of CAMs and ATMs.

SUPPLEMENTARY TABLE S4

Protein Descriptions	Uniprot	Mean ratio 24H Chemerin:Control
Proteasome subunit alpha type-4;Proteasome subunit beta type	P25789;B2RDG0;[illegible]	2.33
Isoform Alpha of Caveolin-1;Caveolin (Fragment);Caveolin;Isofo[illegible]	Q03135-1;Q0313[illegible]	1.90
Macrophage migration inhibitory factor	P14174;A6MUU8	1.84
PDZ and LIM domain protein 5;PDZ and LIM domain 5 isoform b	Q96HC4;B7Z481;[illegible]	1.68
Heat shock 70 kDa protein 1;cDNA FLJ54392, highly similar to H[illegible]	P08107;A8K5I0;B[illegible]	1.65
annexin IV;Annexin A4	Q6LES2;P09525;B[illegible]	1.57
CD151 antigen	P48509;Q53FU5	1.54
Protein disulfide-isomerase A4	P13667;A8K4K6;Q	1.52
Protein DJ-1	Q99497	1.51
Guanine nucleotide-binding protein subunit beta-2-like 1;cDNA [illegible]	P63244;B4DVD2;[illegible]	1.51
Thymosin beta-10	P63313;Q596K9	1.50
Alpha-actinin-4;cDNA FLJ58087, highly similar to Alpha-actinin-4	O43707;Q96BG6;[illegible]	1.50
Ras-related protein Rap-1b-like protein;Ras-related protein Rap-	A6NIZ1;B4DQI8;P[illegible]	1.49
Protein S100-A10	P60903;Q6FGE5	1.49
40S ribosomal protein S3;Ribosomal protein S3	P23396;Q53G83;[illegible]	1.49
Isoform IIb of Prolyl 4-hydroxylase subunit alpha-2;Isoform IIa o[illegible]	O15460-1;O1546[illegible]	1.47
Isoform 1 of Filamin-C;Isoform 2 of Filamin-C	Q14315-1;Q1431[illegible]	1.46
Putative uncharacterized protein ARPC3 (Fragment);Actin-relate	A8MYY8;O15145;[illegible]	1.46
Isocitrate dehydrogenase [NADP] cytoplasmic;cDNA FLJ57227, h	O75874;B2R5M8;[illegible]	1.45
Isoform 1 of 60S ribosomal protein L12;Isoform 2 of 60S ribosom	P30050-1;P30050;	1.45
Putative uncharacterized protein;SH3 domain-binding glutamic [illegible]	Q86Z22;Q9H299;[illegible]	1.45
HSPA5 protein	P11021;B4DEF7;Q	1.45
plasminogen activator inhibitor type 1, member 2 isoform c pre[illegible]	B4DIF2;P07093-1;	1.44
60S ribosomal protein L6;60S ribosomal protein L6;60S ribosom[illegible]	B2R4K7;Q9HBB3;[illegible]	1.44
Synaptic vesicle membrane protein VAT-1 homolog	Q99536;B0AZP7;B	1.44
28 kDa protein;26 kDa protein;Rho GDP-dissociation inhibitor 1;	P52565;B2R5X1;Q	1.44
Calpain-2 catalytic subunit;cDNA FLJ58224, highly similar to Calp	P17655;B2RCM3;[illegible]	1.43
Putative uncharacterized protein RPL7 (Fragment);30 kDa protei	A8MVV7;P18124;[illegible]	1.43
Adenylate kinase 1;Adenylate kinase isoenzyme 1;Adenylate kin	Q53EY8;Q5T9B7;P	1.42
60 kDa heat shock protein, mitochondrial;cDNA FLJ51046, highl[illegible]	P10809;B3GQS7;B	1.42
ubiquitin and ribosomal protein S27a precursor;ubiquitin C;39 k[illegible]	P62979;B2RDW1;[illegible]	1.42
Pituitary tumor-transforming gene 1 protein-interacting protein;	P53801;A8K274;A	1.42
Ubiquitin-like modifier-activating enzyme 1;cDNA FLJ53392, hig[illegible]	P22314;B4DDE4;B	1.42
Cofilin-1;cDNA FLJ51435, moderately similar to Cofilin-1	P23528;B4E112	1.42
Chloride intracellular channel protein 4;cDNA FLJ38640 fis, clon[illegible]	Q9Y696;B4DWC4;	1.42
Isoform 1 of Collagen alpha-3(VI) chain;COL6A3 protein;322 kDa	P12111-1;P12111;	1.41
cDNA FLJ54776, highly similar to Cell division control protein 42	B4E1U9;P60953-2	1.41
Isoform 1 of Brain acid soluble protein 1;Isoform 2 of Brain acid	P80723-1;P80723;	1.41
Moesin;Putative uncharacterized protein MSN (Fragment)	P26038;Q6PJT4;A[illegible]	1.41
Isoform 1 of Microtubule-associated protein 4;Isoform 6 of Mic[illegible]	P27816-1;P27816;	1.41
Serine/threonine-protein phosphatase 2A 65 kDa regulatory su[illegible]	P30153;A8K3H8;A	1.40
Plastin-3;cDNA FLJ59681, highly similar to Plastin-3	P13797;A8K579;B[illegible]	1.40
Malate dehydrogenase;Malate dehydrogenase;Putative unchar[illegible]	P40925;B4DUN2;[illegible]	1.40
Isoform 1 of Tryptophanyl-tRNA synthetase, cytoplasmic;trypto[illegible]	P23381-1;P23381;	1.39
Isoform 1 of Voltage-dependent anion-selective channel protein	P45880-1;P45880;	1.39
40S ribosomal protein S19	P39019;B0ZBD0;Q	1.39
Alpha-crystallin B chain;12 kDa protein;cDNA FLJ57064, highly si	P02511;C3VMY8;[illegible]	1.39

Zyxin;ZYX protein (Fragment);cDNA FLJ53160, highly similar to Z	Q15942;B4DY85;C	1.39
Isoform CD44 of CD44 antigen;Isoform 5 of CD44 antigen;Isofor	P16070-1;P16070;	1.39
Isoform 1 of Plectin-1;Isoform 2 of Plectin-1;Isoform 3 of Plectin	Q15149-1;Q1514S	1.39
fermitin family homolog 2 isoform 2;Isoform 1 of Fermitin family	A8K6S3;B5TJY2;Q!	1.39
14 kDa phosphohistidine phosphatase;phosphohistidine phosph	Q9NRX4;Q6FIE5;B	1.39
Isoform B of Perilipin-3;mannose 6 phosphate receptor binding	O60664-1;O60664	1.38
40S ribosomal protein S28	P62857;B2R4R9	1.38
6-phosphogluconate dehydrogenase, decarboxylating;6-phosph	P52209;A8K2Y9;B	1.37
Peroxiredoxin-2;cDNA FLJ60461, highly similar to Peroxiredoxin-	P32119;B4DF70;A	1.37
Protein S100-A6	P06703;B2R577	1.37
Isoform 1 of Surfeit locus protein 4;cDNA FLJ53092, moderately	O15260-1;O1526C	1.37
Nicotinamide N-methyltransferase	P40261;Q6FH49	1.37
TMSB4X protein (Fragment);Thymosin beta-4-like protein 3;Thy	Q0P5N8;Q0P5P4;(	1.37
HCG1784554, isoform CRA_a;Eukaryotic translation initiation fa	B3KSH1;O00303;B	1.36
cDNA FLJ59142, highly similar to Epididymal secretory protein E	B4DV10;P61916;B	1.36
Isoform 2 of Reticulon-4;Isoform 1 of Reticulon-4;Isoform 5 of R	Q9NQC3-2;Q9NQ(	1.35
Calreticulin	P27797;B4DHR1;B	1.35
Serpin H1;cDNA FLJ52569, highly similar to Collagen-binding pro	P50454;A8K259;Q	1.35
UMP-CMP kinase 1 isoform a;UMP-CMP kinase 1 isoform b;cDN	P30085;B2R6S5;B	1.35
Isoform Mitochondrial of Peroxiredoxin-5, mitochondrial;peroxi	P30044-1;P30044;	1.35
cDNA FLJ56301, highly similar to NADH-cytochrome b5 reductas	P00387-1;P00387;	1.35
Heat shock protein HSP 90-beta	P08238;A8K3W9;E	1.34
Elongation factor 1-gamma;cDNA FLJ56389, highly similar to Elo	P26641;Q2F838;Q	1.34
Isoform 2 of Vinculin;Isoform 1 of Vinculin	P18206-1;P18206;	1.33
Phosphoprotein enriched in astrocytes 15, isoform CRA_b;Astro	B1AKZ3;Q6FHL9;C	1.33
Isoform 1 of 60S ribosomal protein L11;Isoform 2 of 60S ribosom	P62913-1;P62913;	1.33
filamin B isoform 1;Isoform 1 of Filamin-B;filamin B isoform 3;fil	B2ZZ83;O75369-1	1.33
Actin-related protein 3	P61158;B4DT29;B	1.33
40S ribosomal protein S9;RPS9 protein (Fragment);Similar to 40S	P46781;A9C4C1;A	1.33
FK506-binding protein 10;cDNA FLJ53423, highly similar to FK50	Q96AY3;Q658U4;(	1.33
60S acidic ribosomal protein P0;60S acidic ribosomal protein P0-	P05388;A8K4Z4;Q	1.33
Eukaryotic initiation factor 4A-I	P60842;A8K088;A	1.32
Cathepsin B;cDNA FLJ58073, moderately similar to Cathepsin B;(	P07858;A8K2H4;B	1.32
Isoform 2 of Protein disulfide-isomerase A6;Isoform 1 of Protein	Q15084-2;Q15084	1.32
UDP-glucose 6-dehydrogenase;UDP-glucose dehydrogenase, iso	O60701;B4DN25;(	1.32
Isoform 1 of Myoferlin;Isoform 6 of Myoferlin;Isoform 2 of Myo	Q9NZM1-1;Q9NZN	1.32
Isoform 1 of Polymerase I and transcript release factor;cDNA FLJ	Q6NZI2-1;Q6NZI2;	1.32
Protein disulfide-isomerase A3;cDNA FLJ53558, highly similar to	P30101;B3KQT2;B	1.32
Isoform 1 of Prolyl 4-hydroxylase subunit alpha-1;Isoform 2 of P	P13674-1;P13674;	1.32
ADP-ribosylation factor 4;Putative uncharacterized protein ARF4	P18085;C9JPM4;C	1.31
Isoform 1 of Heat shock cognate 71 kDa protein;54 kDa protein;	P11142-1;P11142;	1.31
Isoform 2 of Tropomyosin alpha-3 chain;tropomyosin 3 isoform	P06753-2;P06753;	1.31
Isoform 2 of Heat shock protein HSP 90-alpha;Isoform 1 of Heat	P07900-2;P07900;	1.31
Ras GTPase-activating-like protein IQGAP1	P46940;A4QPB0;B	1.31
D-3-phosphoglycerate dehydrogenase;Phosphoglycerate dehydr	O43175;B3KSC3;Q	1.30
actinin, alpha 1 isoform a;Alpha-actinin-1;actinin, alpha 1 isofor	A1L0V1;B4DHH3;(	1.30
14-3-3 protein epsilon;22 kDa protein	P62258;B7ZA86;Q	1.30
45 kDa protein;Fructose-bisphosphate aldolase A	P04075;A4UCS9;A	1.30
Isoform M2 of Pyruvate kinase isozymes M1/M2;66 kDa protein	P14618-1;P14618;	1.30
Endoplasmin;cDNA FLJ58626, highly similar to Endoplasmin	P14625;Q59FC6;Q	1.30

Coatomer subunit gamma	Q9Y678;A8K6M8;I	1.30
Isoform 1 of F-actin-capping protein subunit beta;Isoform 2 of F-	P47756-1;P47756;	1.30
Neuroblast differentiation-associated protein AHNAK;Putative u	Q09666;B4DTV0;C	1.30
Isoform 2 of Procollagen-lysine,2-oxoglutarate 5-dioxygenase 2;	O00469-2;O00469	1.30
Talin-1	Q9Y490;Q5TCU6	1.30
Isoform 1 of Clathrin heavy chain 1;Isoform 2 of Clathrin heavy c	Q00610-1;Q00610	1.29
Protein disulfide-isomerase;cDNA FLJ59430, highly similar to Prc	P07237;B2RDQ2;E	1.29
Myristoylated alanine-rich C-kinase substrate	P29966;Q05C82;C	1.29
Glycogen phosphorylase, brain form	P11216;B4DSD8;C	1.29
Peptidyl-prolyl cis-trans isomerase B;20 kDa protein	P23284	1.29
Isoform 2 of Annexin A2;Isoform 1 of Annexin A2;Putative anne>	P07355-2;P07355;	1.29
Glutathione S-transferase omega-1;Glutathione S-transferase or	P78417;B2R983;Q	1.29
Myosin regulatory light chain MRCL3 variant;Myosin regulatory	Q53HL1;O14950;F	1.29
S-formylglutathione hydrolase;28 kDa protein;Putative unchara(	P10768;C9J3H9	1.29
Destrin;destrin isoform b;Pseudogene candidate;hypothetical pr	P60981;B4DYA6;B	1.29
21 kDa protein;Calmodulin;cDNA FLJ75174, highly similar to Hor	P62158;B4DJ51;A8	1.28
Isoform 2 of Eukaryotic translation initiation factor 5A-1;Isoform	P63241-2;P63241;	1.28
Peroxiredoxin-1;19 kDa protein	Q06830;B2R4P2;B	1.28
Phosphatidylethanolamine-binding protein 1;22 kDa protein;cDI	P30086;B4DRT4	1.28
Nucleoside diphosphate kinase;Isoform 3 of Nucleoside diphosp	Q32Q12;P22392-2	1.28
Phosphoglycerate mutase 1;29 kDa protein	P18669;B4DJA4;B4	1.28
cDNA FLJ31776 fis, clone NT2RI2008141, highly similar to CALUI	B3KPG9;B3KQF5;C	1.28
Annexin A1;Annexin A1	P04083;B5BU38;C	1.28
ATP synthase subunit beta, mitochondrial	P06576;Q0QEN7	1.28
Elongation factor 1-alpha 1;Putative elongation factor 1-alpha-lil	P68104;A8K9C4;A	1.28
Heat shock protein beta-1;cDNA FLJ52243, highly similar to Heat	P04792;B4DL87;C9	1.28
Isoform 2 of Septin-2;Isoform 1 of Septin-2;Putative uncharacter	Q15019-2;Q15019	1.27
14-3-3 protein zeta/delta	P63104;D0PNI1	1.27
Isoform 1 of Phosphoglucomutase-1;Isoform 2 of Phosphogluco	P36871-1;P36871;	1.27
Prosaposin;Isoform Sap-mu-0 of Proactivator polypeptide;Isofor	B1AVU8;P07602-1	1.27
Elongation factor 2;cDNA FLJ56548, highly similar to Elongation	P13639;B4DMC6;I	1.27
L-lactate dehydrogenase B chain;L-lactate dehydrogenase;Putati	P07195;Q5U077;A	1.27
FSCN1 protein (Fragment);Fascin	Q96IH1;Q16658;B	1.27
programmed cell death 6 interacting protein isoform 2;Program	B4DHD2;B7Z5C1;C	1.27
Dolichyl-diphosphooligosaccharide--protein glycosyltransferase	P04844;B2RE46;Q	1.27
Chloride intracellular channel protein 1	O00299;Q53FB0;C	1.27
40S ribosomal protein S8;Ribosomal protein S8	P62241;Q5JR94;Q	1.27
Reticulocalbin-3	Q96D15	1.26
Annexin A5;Putative uncharacterized protein ANXA5 (Fragment)	P08758;B4DNG6;C	1.26
Phosphoglycerate kinase 1;Phosphoglycerate kinase;Phosphogly	P00558;A8K4W6;E	1.26
Peptidyl-prolyl cis-trans isomerase A;cDNA FLJ53060, moderatel	P62937;A8K220;A	1.26
Isoform 1 of Adenylyl cyclase-associated protein 1;Isoform 2 of /	Q01518-1;Q01518	1.26
Transitional endoplasmic reticulum ATPase	P55072;Q0IIN5;Q9	1.26
Isoform 1 of Protein-glutamine gamma-glutamyltransferase 2;Is	P21980-1;P21980;	1.26
Isoform 1 of Tropomyosin alpha-4 chain;Isoform 2 of Tropomyo:	P67936-1;P67936;	1.26
CD59 glycoprotein	P13987;Q6FHM9	1.26
EH domain-containing protein 2;cDNA FLJ39909 fis, clone SPLEN	Q9NZN4;B4DLA1;I	1.26
Isoform 1 of Nucleophosmin;Isoform 2 of Nucleophosmin;nucle	P06748-1;P06748;	1.25
Guanine nucleotide-binding protein G(I)/G(S)/G(T) subunit beta-	P62873;B2R6K4;B	1.25
p180/ribosome receptor;Isoform 3 of Ribosome-binding protein	A7BI36;Q9P2E9-1;	1.25

60S ribosomal protein L27a	P46776;Q6NZ52;C	1.25
cDNA FLJ56329, highly similar to Myosin light polypeptide 6;Isof	B7Z6Z4;P60660-1;	1.25
60S ribosomal protein L4;cDNA FLJ50996, highly similar to 60S ri	P36578;B4DFI6;Q5	1.25
Ribonuclease inhibitor	P13489;Q59GB3;C	1.25
cDNA FLJ56442, highly similar to ATP-citrate synthase;ATP-citrat	B4DIM0;Q4LE36;P	1.25
Isoform Long of Glucose-6-phosphate 1-dehydrogenase;Isoform	P11413-2;P11413;	1.25
cDNA FLJ53193, highly similar to Homo sapiens caldesmon 1 (CA	Q05682-4;Q05682	1.25
Isoform 1 of Filamin-A;Isoform 2 of Filamin-A;Filamin A, alpha;Pı	P21333-1;P21333;	1.25
Transgelin;Transgelin variant	Q01995;Q53GC9;(	1.25
Tubulin alpha-1A chain;Tubulin alpha-1C chain;cDNA FLJ55956, I	Q71U36;A8K0B8;(	1.25
Phosphoserine aminotransferase;45 kDa protein;Isoform 1 of Pł	B4DHQ3;Q9Y617-1	1.24
Glutathione S-transferase P;Putative uncharacterized protein GS	P09211;C7DJS1;C7	1.24
Isoform alpha-enolase of Alpha-enolase;Isoform MBP-1 of Alpha	P06733-1;P06733;	1.24
24 kDa protein;Transgelin-2;21 kDa protein	P37802	1.24
Protein S100-A11	P31949;B2R5H0	1.24
Isoform 1 of Myosin-9;Isoform 2 of Myosin-9	P35579-1;P35579;	1.24
Tubulin beta-2C chain;cDNA FLJ11352 fis, clone HEMBA1000020	P68371;Q8IWP6;C	1.24
40S ribosomal protein S2;31 kDa protein;Putative characterize	P15880;A4D0Y7;C	1.24
Annexin A6;annexin VI isoform 2	P08133;A8K3Q7;B	1.24
Isoform 1 of Thioredoxin reductase 1, cytoplasmic;Isoform 6 of T	Q16881-1;Q16881	1.23
Ubiquitin-conjugating enzyme E2 N;Ubiquitin carrier protein (Fra	P61088;A8MUJ2;C	1.23
14-3-3 protein gamma;cDNA FLJ52141, highly similar to 14-3-3 p	P61981;B3KNB4;B	1.23
Voltage-dependent anion-selective channel protein 1;Similar to	P21796;B3KTS5;B4	1.23
Glucose-6-phosphate isomerase;Glucose-6-phosphate isomerase	B4DE36;B4DG39;F	1.23
Galectin-1	P09382;B2R5E8;Q	1.23
Glyceraldehyde-3-phosphate dehydrogenase;32 kDa protein;Gly	P04406;A4UCT1;C	1.23
Dihydropyrimidinase-related protein 2;Dihydropyrimidinase-like	Q16555;A8K5H2;A	1.23
Cathepsin D;Putative uncharacterized protein CTSD;Putative unc	P07339;C9JIH9;C9	1.22
33 kDa protein;Putative uncharacterized protein RPSAP58;40S ri	A6NE09;P08865;C	1.22
Isoform 1 of Cytoskeleton-associated protein 4;Isoform 2 of Cyto	Q07065-1;Q07065	1.22
Glycyl-tRNA synthetase;Putative uncharacterized protein GARS	P41250;Q75MN1	1.22
Aminopeptidase N	P15144;B4DP01;B	1.22
Tubulin, beta;cDNA FLJ56903, highly similar to Tubulin beta-7 ch	B4DQN9;B7ZAF0;E	1.22
Thioredoxin domain-containing protein 5;Putative uncharacteriz	Q8NBS9;Q658S9;C	1.21
Peroxiredoxin-6	P30041;A4UCS6	1.21
Putative uncharacterized protein ARPC4;Putative uncharacterize	C9JWM7;C9J0N8;I	1.21
Profilin-1	P07737;Q53Y44	1.21
T-complex protein 1 subunit gamma;chaperonin containing TCP1	P49368;B4DUR8;C	1.21
lactate dehydrogenase A isoform 3;Isoform 1 of L-lactate dehydı	B7Z5E3;P00338-1;	1.21
Isoform 15 of Fibronectin;Isoform 7 of Fibronectin;fibronectin 1	P02751-15;P02751	1.21
40S ribosomal protein S12	P25398	1.21
14-3-3 protein eta;Homo sapiens clone CDABP0046 mRNA sequ	Q04917;B2R6N6;A	1.21
ADP-ribosylation factor 1;ADP-ribosylation factor 3;cDNA FLJ61C	P84077;P61204;B4	1.21
cDNA FLJ60299, highly similar to Rab GDP dissociation inhibitor I	B4DLV7;Q5SX90;P	1.20
Lysosome membrane protein 2	Q14108;Q53Y63	1.20
actin-related protein 2 isoform a;Actin-related protein 2;cDNA F	B4DHK9;P61160;C	1.20
Major vault protein;cDNA FLJ53437, highly similar to Major vaul	Q14764;B4DDR2;E	1.20
Isoform Long of 14-3-3 protein beta/alpha;Isoform Short of 14-3	P31946-1;P31946;	1.20
Isoform 3 of Tropomyosin alpha-1 chain;tropomyosin 1 alpha ch	P09493-3;P09493;	1.19
ATP synthase subunit alpha, mitochondrial;ATP synthase subuni	P25705;A8K092;B4	1.19

CD63 antigen;CD63 antigen isoform B;Lysosome-associated mer	P08962;C9JV86;Q	1.19
cDNA FLJ52765, highly similar to Calponin-2;Calponin-2;cDNA FL	B4DUT8;Q99439;	1.19
Tumor protein, translationally-controlled 1;Translationally-contr	Q5W0H4;P13693;	1.19
cDNA FLJ52712, highly similar to Tubulin beta-6 chain;46 kDa pr	B4DP54;B4E386	1.19
Putative uncharacterized protein WDR1;Isoform 1 of WD repeat	A8MVA9;Q53GN4	1.19
triosephosphate isomerase 1 isoform 2;Isoform 1 of Triosephos	Q53HE2;P60174-1	1.19
14-3-3 protein theta;Putative uncharacterized protein YWHAQ	P27348;B4DMT8;	1.18
Calpain small subunit 1	P04632	1.18
Cystatin-B	P04080;Q76LA1	1.18
Collapsin response mediator protein 4 long variant;Dihydropyrin	B3SXQ8;Q6DEN2;	1.17
Vimentin	P08670;B0YJC4;B0	1.17
Serine/threonine protein phosphatase;protein phosphatase 1, c	B3KXM2;Q07161;	1.17
Cysteine and glycine-rich protein 1;cysteine and glycine-rich pro	P21291;A8K268;B	1.17
Actin-related protein 2/3 complex subunit 2;PNAS-139	O15144;Q53R19;	1.17
Stress-70 protein, mitochondrial;cDNA FLJ51903, highly similar t	P38646;B7Z1V7;B	1.16
Malate dehydrogenase, mitochondrial;cDNA FLJ52880, highly si	P40926;Q0QF37;	1.16
Isoform Beta-1A of Integrin beta-1;Isoform Beta-1C of Integrin b	P05556-1;P05556;	1.16
Isoform 1 of Ras-related protein Rab-1A;23 kDa protein;cDNA FL	P62820-1;P62820;	1.16
Lysosome-associated membrane glycoprotein 1;cDNA FLJ57633,	P11279;B3KRY3;B	1.15
Tropomyosin 2;Tropomyosin 2;Isoform 1 of Tropomyosin beta c	Q5TCU3;Q5TCU8;	1.15
HCG2042771;Tubulin beta-3 chain;HCG1983504, isoform CRA_f;	Q01726;A8I0M1;B	1.14
Isoform 1 of LIM and SH3 domain protein 1;Isoform 2 of LIM an	Q14847-1;Q14847	1.14
Actin, alpha skeletal muscle;Actin, alpha cardiac muscle 1;cDNA	P68133;P68032;A	1.12
cDNA FLJ54957, highly similar to Transketolase;Transketolase;tr	B4DE31;P29401;A	1.12
Collagen alpha-1(I) chain	P02452;Q14042;C	1.12
40S ribosomal protein S4, X isoform;13 kDa protein	P62701;B2R491;Q	1.11
BTB/POZ domain-containing protein KCTD12	Q96CX2;B3KY04	1.10
Collagen alpha-1(VI) chain	P12109;B4DRR8;C	1.10
40S ribosomal protein S5	P46782;Q53G25	1.10
Thioredoxin-dependent peroxide reductase, mitochondrial;pero	P30048;A4UCS5;C	1.10
Ezrin;69 kDa protein	P15311;B2R6J2;B	1.05
Isoform 2 of Heterogeneous nuclear ribonucleoprotein K;Isoforr	P61978-2;P61978;	1.05
cDNA FLJ56293, highly similar to Transmembrane glycoprotein N	B4E3D4;Q14956-1	1.03
NAD(P)H dehydrogenase [quinone] 1;NQO1 protein (Fragment);	P15559;Q53G81;B	1.01
FHL2 isoform 5;Four and a half LIM domains protein 2	Q2I5I4;Q2TSB7;Q	1.00
Isoform A of Lamin-A/C;Isoform ADelta10 of Lamin-A/C;Progerir	P02545-1;P02545;	0.95

Supplementary Table S4. Proteins identified in cell extracts of SILAC labelled MSCs subsequently treated with chemerin for 24 h. Identifications restricted to proteins recorded in 3 of 3 replicates on the basis of at least two tryptic peptides. Identification of MIF is highlighted.

SUPPLEMTARY TABLE S5

Protein Names	Gene Names	Protein Descriptions	Uniprot	PEP	Chemerin/ Control
RAR-responsive protein TI	RARRES2;TIG2;hC	Retinoic acid receptor responder protein 2;Putative uncharacteri	Q99969;Q7LE02;C9J	2.69E-14	24.42
ACAN protein;Aggrecan co	ACAN;AGC1;CSPG	ACAN protein;250 kDa protein;Isoform 1 of Aggrecan core protei	B9EK55;P16112-1;P	1.06E-21	5.93
Collagen-binding 59 kDa p	FM;FMOD;SLRR2	Fibromodulin;cDNA FLJ35580 fis, clone SPLEN2006389, highly sin	Q06828;B4E1J3;B3K	7.88E-20	3.26
Actin-depolymerizing fact	GSN;hCG_27454;	Isoform 1 of Gelsolin;gelsolin isoform c;Isoform 2 of Gelsolin;cDN	P06396-1;P06396;B	5.09E-48	2.39
CDw108;JMH blood group	CD108;SEMA7A;S	Semaphorin-7A;semaphorin 7A isoform 2 preproprotein	O75326;B4DDP7	1.97E-18	2.10
Calumenin, isoform CRA_c	CALU;hCG_41656	cDNA FLJ31776 fis, clone NT2RI2008141, highly similar to CALUM	B3KPG9;B3KQF5;O4	1.27E-09	1.88
Biglycan;Bone/cartilage pr	BGN;SLRR1A	Biglycan;Putative uncharacterized protein BGN;cDNA FLJ35704 fi	P21810;A8K7E0;B4D	1.52E-21	1.71
Collagen alpha-1(V) chain;	COL5A1	Collagen alpha-1(V) chain	P20908;B2ZZ86;Q5	8.65E-24	1.56
Membrane-organizing ext	MSN	Moesin;Putative uncharacterized protein MSN (Fragment);cDNA	P26038;Q6PJT4;A8N	1.19E-10	1.55
Collagen alpha-3(VI) chain	COL6A3;DKFZp68	Isoform 1 of Collagen alpha-3(VI) chain;COL6A3 protein;322 kDa	P12111-1;P12111;B	1.50E-132	1.55
Collagen alpha-2(VI) chain	COL6A2	Isoform 2C2 of Collagen alpha-2(VI) chain;Isoform 2C2A of Collag	P12110-1;P12110;Q	1.64E-41	1.50
Collagen alpha-1(VI) chain	COL6A1	Collagen alpha-1(VI) chain	P12109;B4DRR8;Q0	1.22E-187	1.48
Cartilage oligomeric matri	COMP	Cartilage oligomeric matrix protein;cDNA FLJ60724, highly simila	P49747;A8K3I0;B4D	1.58E-79	1.47
L-lactate dehydrogenase;C	LDHA;PIG19;hCG_	lactate dehydrogenase A isoform 3;Isoform 1 of L-lactate dehydr	B7Z5E3;P00338-1;P	1.18E-36	1.46
Alcadein-alpha;Alzheimer-	CLSTN1;CS1;KIAA	Isoform 1 of Calsyntenin-1 (Fragment);Isoform 2 of Calsyntenin-1	O94985-1;O94985;C	4.37E-06	1.43
Collagen alpha-1(XII) chain	COL12A1;COL12A	Isoform 1 of Collagen alpha-1(XII) chain;Isoform 4 of Collagen alp	Q99715-1;Q99715;E	1.44E-159	1.43
Cytosolic thyroid hormone	OIP3;PK2;PK3;PKI	66 kDa protein;Isoform M1 of Pyruvate kinase isozymes M1/M2;	P14618-2;P14618;P	5.88E-36	1.40
Actin-binding protein 280;	FLN;FLN1;FLNA;Fl	Isoform 1 of Filamin-A;Isoform 2 of Filamin-A;Filamin A, alpha;Pu	P21333-1;P21333;Q	6.09E-86	1.40
Caldesmon;cDNA FLJ7664	CAD;CALD1;CDM;	Isoform 1 of Caldesmon;Isoform 2 of Caldesmon;caldesmon 1 isc	Q05682-1;Q05682;C	1.18E-09	1.37
Annexin A2;Annexin II;Ann	ANX2;ANX2L4;AN	Isoform 2 of Annexin A2;Isoform 1 of Annexin A2;24 kDa protein;	P07355-2;P07355;P	5.01E-24	1.37
Collagen alpha-2(V) chain;	COL5A2	Collagen alpha-2(V) chain;110 kDa protein;COL5A2 protein	P05997;B4DNJ0;Q5	1.73E-17	1.36
2-phospho-D-glycerate hy	ENO1;ENO1L1;MI	Isoform alpha-enolase of Alpha-enolase;Isoform MBP-1 of Alpha-	P06733-1;P06733;A	6.18E-20	1.36
Anastellin;Cold-insoluble g	FN;FN1;DKFZp68	Isoform 1 of Fibronectin;Isoform 3 of Fibronectin;Isoform 15 of F	P02751-1;P02751;A	0	1.36
Procollagen C-endopeptid	PCOLCE;PCPE1;tc	Procollagen C-endopeptidase enhancer 1;cDNA FLJ55126, highly	Q15113;A4D2D2;B4	1.47E-13	1.36
ABI gene family member 3	ABI3BP;NESHBP;T	Target of Nesh-SH3 precursor (Tarsh) (Nesh-binding protein) (Ne	Q7Z7G0-3;Q7Z7G0;	7.28E-13	1.36
Erythroid-potentiating act	CLGI;TIMP;TIMP1	Metalloproteinase inhibitor 1;TIMP metallopeptidase inhibitor 1;	P01033;B3KQF4;Q5	5.66E-56	1.34
Alpha-1 type I collagen;Co	COL1A1	Collagen alpha-1(I) chain	P02452;Q14042;Q6	0	1.34
14 kDa laminin-binding pr	LGALS1;HL14	Galectin-1	P09382;B2R5E8;Q1	1.24E-17	1.34
Insulin-like growth factor-	BP2;IBP2;IGFBP2	Insulin-like growth factor-binding protein 2;Putative uncharacter	P18065;C9JMY1	8.85E-89	1.33

Collagen alpha-1(III) chain;	COL3A1	Isoform 1 of Collagen alpha-1(III) chain;Isoform 2 of Collagen alpl	P02461-1;P02461;P	8.97E-75	1.32
Latent-transforming growt	C14orf141;LTBP2	Latent-transforming growth factor beta-binding protein 2	Q14767;Q59EE6;Q6	2.00E-24	1.31
FN1 protein	FN1	fibronectin 1 isoform 2 preproprotein	Q14328	0	1.31
Alpha-2 type I collagen;Co	COL1A2	Collagen alpha-2(I) chain	P08123;A2TIK1;Q75	0	1.30
C1 esterase;Complement	C1S	Complement C1s subcomponent;Putative uncharacterized protei	P09871;A8K2N0;B3I	6.36E-23	1.28
Extracellular matrix protei	ECM1	Isoform 4 of Extracellular matrix protein 1;Isoform 1 of Extracellu	Q16610-4;Q16610;	3.30E-15	1.27
Fibrillin-1;cDNA FLJ61597,	FBN;FBN1	Fibrillin-1	P35555;B4E3I6;Q75	4.55E-198	1.27
Bone proteoglycan II;Deco	DCN;SLRR1B;DKF	Isoform A of Decorin;Isoform D of Decorin	P07585-1;P07585;Q	2.39E-22	1.27
Actin, cytoplasmic 2;Actin,	ACTB;ACTG;ACTG	Actin, cytoplasmic 2;cDNA FLJ57283, highly similar to Actin, cyto	P63261;B4E3A4	1.21E-206	1.27
Integrin beta-like protein 1	ITGBL1;OSCP;TIE	Integrin beta-like protein 1;Osteoblast specific cysteine-rich prot	O95965;O14549;B3	3.93E-41	1.25
EGF-containing fibulin-like	EFEMP1;FBLN3;F	Isoform 1 of EGF-containing fibulin-like extracellular matrix prote	Q12805-1;Q12805;	3.00E-83	1.25
Insulin-like growth factor-I	IBP4;IGFBP4	Insulin-like growth factor-binding protein 4	P22692;B4E351	1.67E-12	1.25
Canstatin;Collagen alpha-2	COL4A2	Collagen alpha-2(IV) chain;168 kDa protein	P08572;B4DH43	4.07E-36	1.23
Insulin-like growth factor-I	IBP3;IGFBP3;hCG_	insulin-like growth factor binding protein 3 isoform a precursor;I	P17936;A6XND0;A6	2.87E-23	1.23
Insulin-like growth factor-I	IBP6;IGFBP6	Insulin-like growth factor-binding protein 6	P24592;Q9H2B5	3.55E-09	1.23
Thrombospondin-2;Putati	THBS2;TSP2;DKFZ	Thrombospondin-2;Thrombospondin 2	P35442;Q6MZL6;Q5	3.27E-145	1.22
IGFBP-rP1;Insulin-like grov	IGFBP7;MAC25;P	Insulin-like growth factor-binding protein 7	Q16270;B4E1N2;B7	2.00E-245	1.22
72 kDa gelatinase;72 kDa t	CLG4A;MMP2	72 kDa type IV collagenase;matrix metalloproteinase 2 isoform b	P08253;B4DWH3;Q	4.72E-91	1.22
Kerato-epithelin;RGD-cont	BIGH3;TGFBI;hCG	Transforming growth factor-beta-induced protein ig-h3;60 kDa p	Q15582;Q53GU8;Q	2.19E-119	1.22
CSC-21K;Metalloproteinas	TIMP2;hCG_9557	Metalloproteinase inhibitor 2;22 kDa protein;cDNA FLJ57920, hig	P16035;B4DFW2	4.25E-22	1.20
Vimentin;Vimentin variant	VIM	Vimentin	P08670;B0YJC4;B0Y	5.79E-68	1.19
Basement membrane-spe	HSPG2	Basement membrane-specific heparan sulfate proteoglycan core	P98160;B6EU51;Q2	9.59E-120	1.19
Follistatin-like protein 1;F	FRP;FSTL1	Follistatin-related protein 1;cDNA FLJ50214, highly similar to Foll	Q12841;A8K523;B4	1.28E-114	1.18
Lysyl oxidase;Protein-lysin	LOX;hCG_37363	Protein-lysine 6-oxidase	P28300;B0AZT2;B4D	1.45E-152	1.18
Keratan sulfate proteoglyc	LDC;LUM;SLRR2D	Lumican	P51884;Q53FV4	5.75E-23	1.18
CCN family member 2;Con	CCN2;CTGF;HCS2	Isoform 1 of Connective tissue growth factor;Isoform 2 of Conne	P29279-1;P29279;B	8.76E-36	1.15
Thrombospondin-1;THBS1	THBS1;TSP;TSP1	Thrombospondin-1	P07996;A0PJG0;B4E	0	1.10
Coiled-coil domain-contai	CCDC80;DRO1;HE	Isoform 2 of Coiled-coil domain-containing protein 80;Isoform 1	Q76M96-2;Q76M96	2.22E-13	1.08
Quiescin Q6;Sulfhydryl oxi	QSCN6;QSOX1;U	Isoform 1 of Sulfhydryl oxidase 1;Putative uncharacterized protei	O00391-1;O00391;	3.88E-42	0.95
Cystatin-3;Cystatin-C;Gam	CST3	Cystatin-C	P01034	4.90E-13	0.85
Insulin-like growth factor-I	IBP5;IGFBP5	Insulin-like growth factor-binding protein 5;Putative uncharacter	P24593;C9JXX4	3.98E-10	0.84

Supplementary Table S5. Proteins identified in the media of SILAC labelled MSCs subsequently treated with chemerin for 24 h. Identifications limited to proteins found in 3 of 3 replicates and based on at least two tryptic peptides. Ratio of relative abundance in treated *vs* untreated cells is shown. PEP, probabilty of correct identification. MMP-2 is highlighted.
