# Supplementary material for: Increased Expression of Chemerin in Squamous Esophageal Cancer Myofibroblasts and Role in Recruitment of Mesenchymal Stromal Cells
Source: PLoS One. 2014 Aug 15;9(8):e104877. doi: 10.1371/journal.pone.0104877 (PMC4134237; doi:10.1371/journal.pone.0104877)
Supplement: File S2 — Supplementary Figures. Figure S1 to Figure S10. (PDF) [file pone.0104877.s003.pdf]

Supplementary Figure S1.

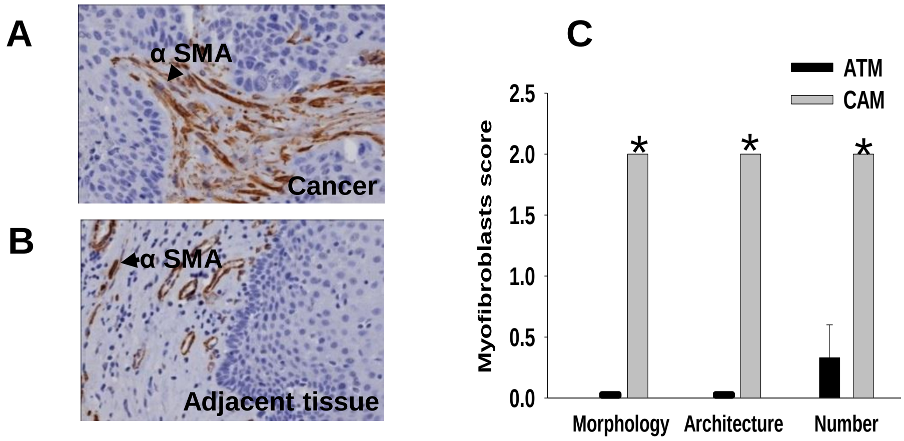

Supplementary Figure S1. α-SMA staining identified myofibroblasts in increased numbers, and with disordered morphology in esophageal squamous cancer. *A,* Myofibroblast morphology in a esophageal squamous cancer (CAM) and *B* in adjacent tissue (ATM) from the same patient. Arrows indicate α-SMA positive myofibroblasts (brown). *C,* Quantification of myofibroblast morphology, architecture and number in cancer, and adjacent tissue. The scoring system for myofibroblast morphology was: 0, normal; 1, mildly distorted; 2, severely distorted. Myofibroblast architecture was scored: 0, restricted to periglandular or subepithelial localisation; 1, both in periglandular/subepithelial regions and elsewhere in the interstitium; 2, severe architectural damage with meshwork-like appearance. Myofibroblast number was scored: 0, as control; 1, mild to moderately increased; 2 substantially increased. For the histopathological assessment, myofibroblasts were defined as stellate/spindle-shaped cells with α-SMA expresssion. Smooth muscle fibers were excluded based on their characteristic morphology. * $p<0.05$.

Supplementary Figure S2.

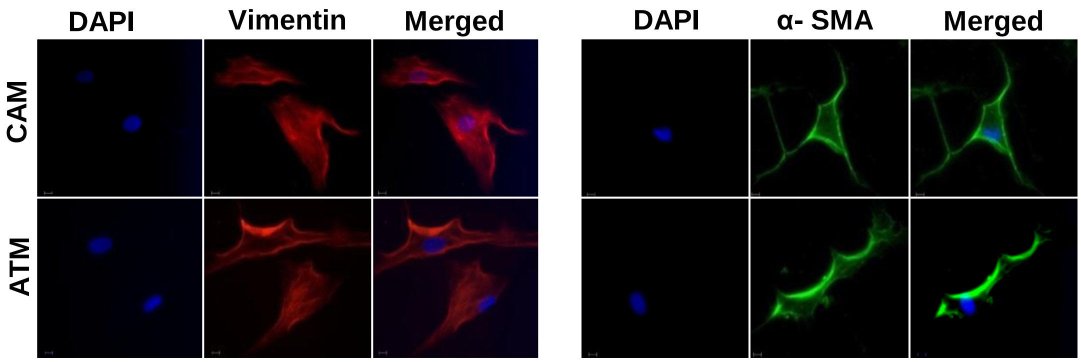

Supplementary Figure S2. Cultured squamous esophageal cancer associated-myofibroblasts (CAMs, top) and adjacent tissue myofibroblasts (ATMs, bottom) exhibited positive staining for α-SMA and vimentin.

Supplementary Figure S3.

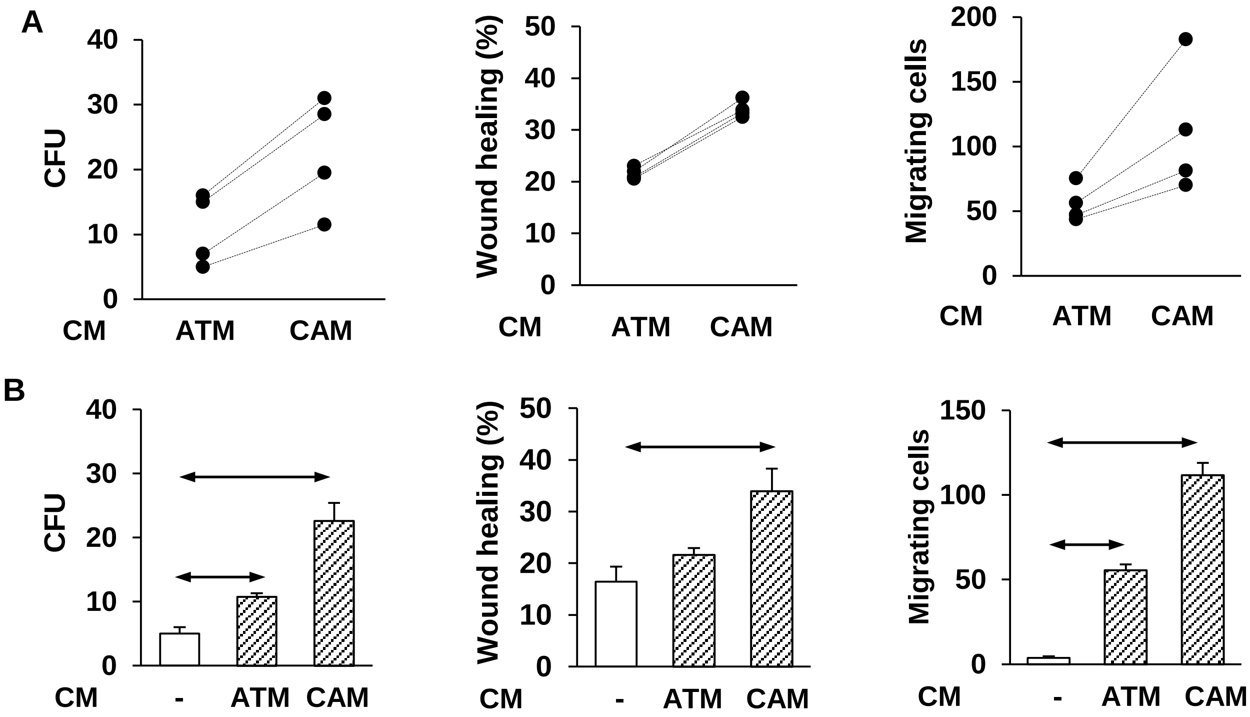

Supplementary Figure S3. CAMs stimulate an OE21 cell growth and migration. *A,* Conditioned media from CAMs increased OE21 cell proliferation as determined in CFU (left) assays and migration as determined in wound healing (middle) and Boyden chamber (right) assays compared to ATM-CM for four paired samples. *B,* Group mean data for the experiments shown in panel *A*. n=4 pairs of CAMs and ATMs from patients described in Supplementary Table 1; horizontal arrows mean $p<0.05$ by ANOVA; bars, SEM.

Supplementary Figure S4.

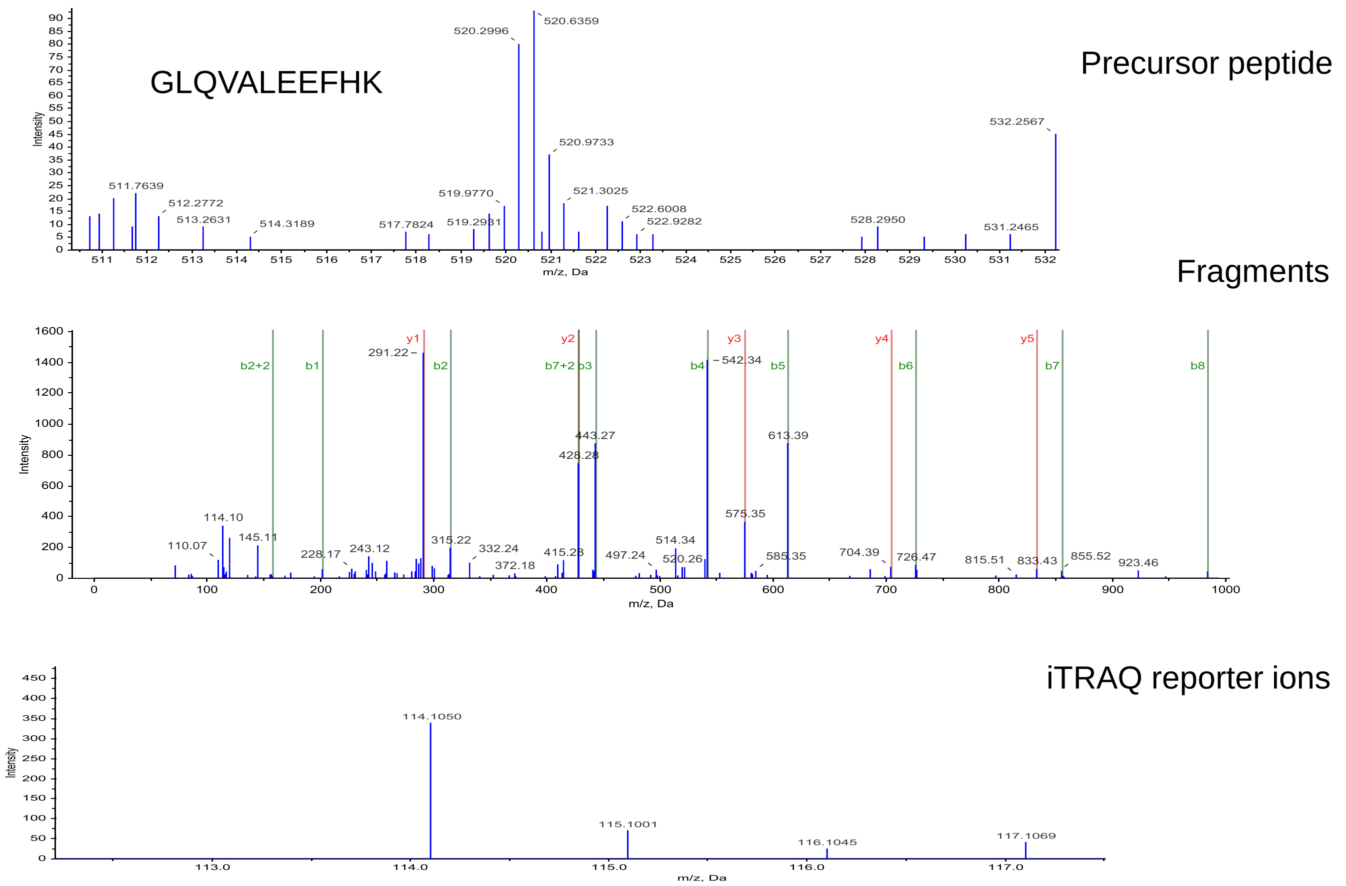

MRRLLIPLAL WLGAVGVGVA ELTEAQRRGL QVALEEFHKH PPVQWAFQE
SVESAVDTPF PAGIFVRLEF KLQQTSCRKR DWKKPECKVR PNGRKRKCLA
CIKLGSEDKV LGRLVHCPIE TQVLREAEEH QETQCLRVQR AGEDPHSFYF
PGQFAFSKAL PRS

Supplementary Figure S4. Identification of chemerin in the media of myofibroblasts. Spectra corresponding to the precursor peptide (GLQVALEEFHK), fragments and iTRAQ reporter ions are shown, together with the aminoacid sequence of preprochemerin indicating tryptic peptides identified by iTRAQ. Peptides in red identified in all subjects; peptides in green identified in one subject.

Supplementary Figure S5.

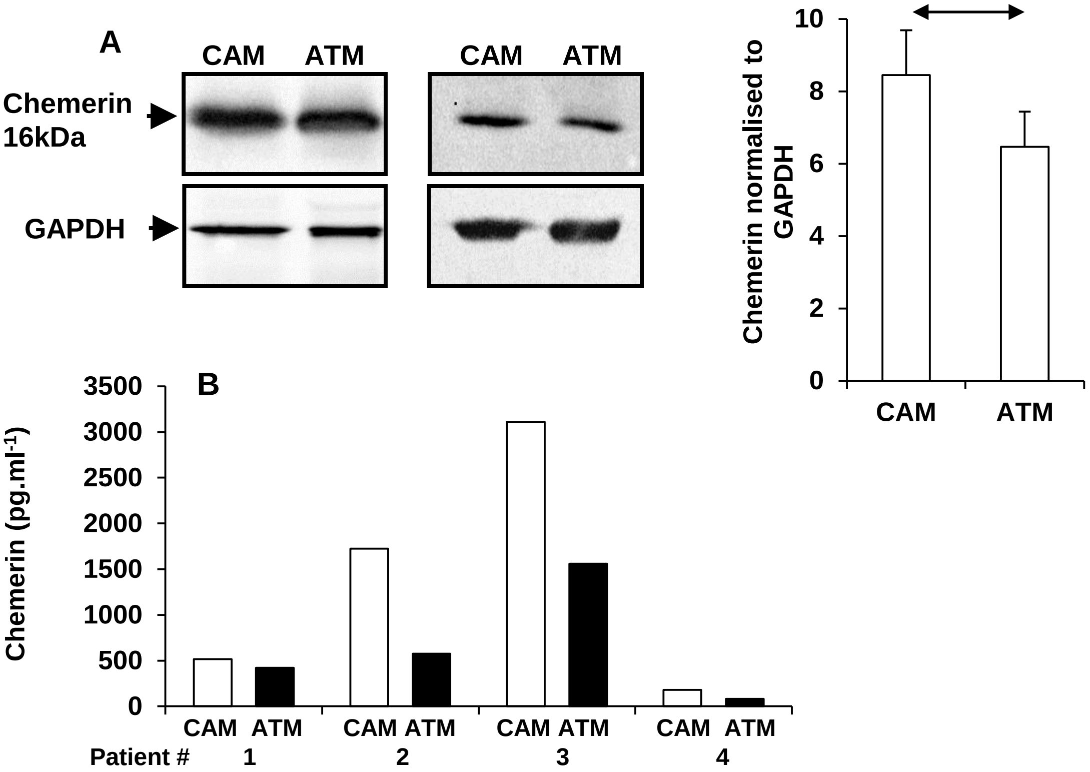

Supplementary Figure S5. Chemerin in CAMs and ATMs. *A*, left, chemerin detected by Western blot in two pairs of CAMs and ATMs (top) and GAPDH in the same samples (bottom). Right, relative abundance of chemerin to GAPDH determined by densitometry after Western blot in four pairs of ESCC CAMs and ATMs; horizontal arrow, $p<0.05$, paired t test. *B,* abundance of chemerin determined by ELISA in media of pairs of CAMs and ATMs from four patients (see Supplementary Table 1 for information on each patient).

Supplementary Figure S6.

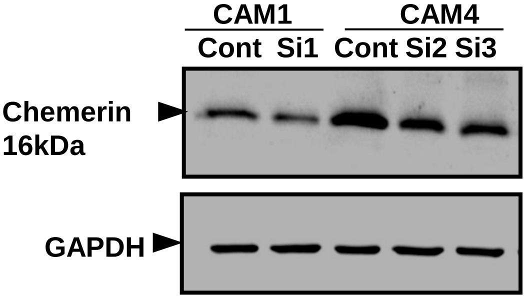

Supplementary Figure S6. Western blots showing chemerin knock-down in siRNA treated CAMs; two CAMs are shown - Si1 in CAM1, and Si2 and Si3 CAM4 cells.

Supplementary Figure S7.

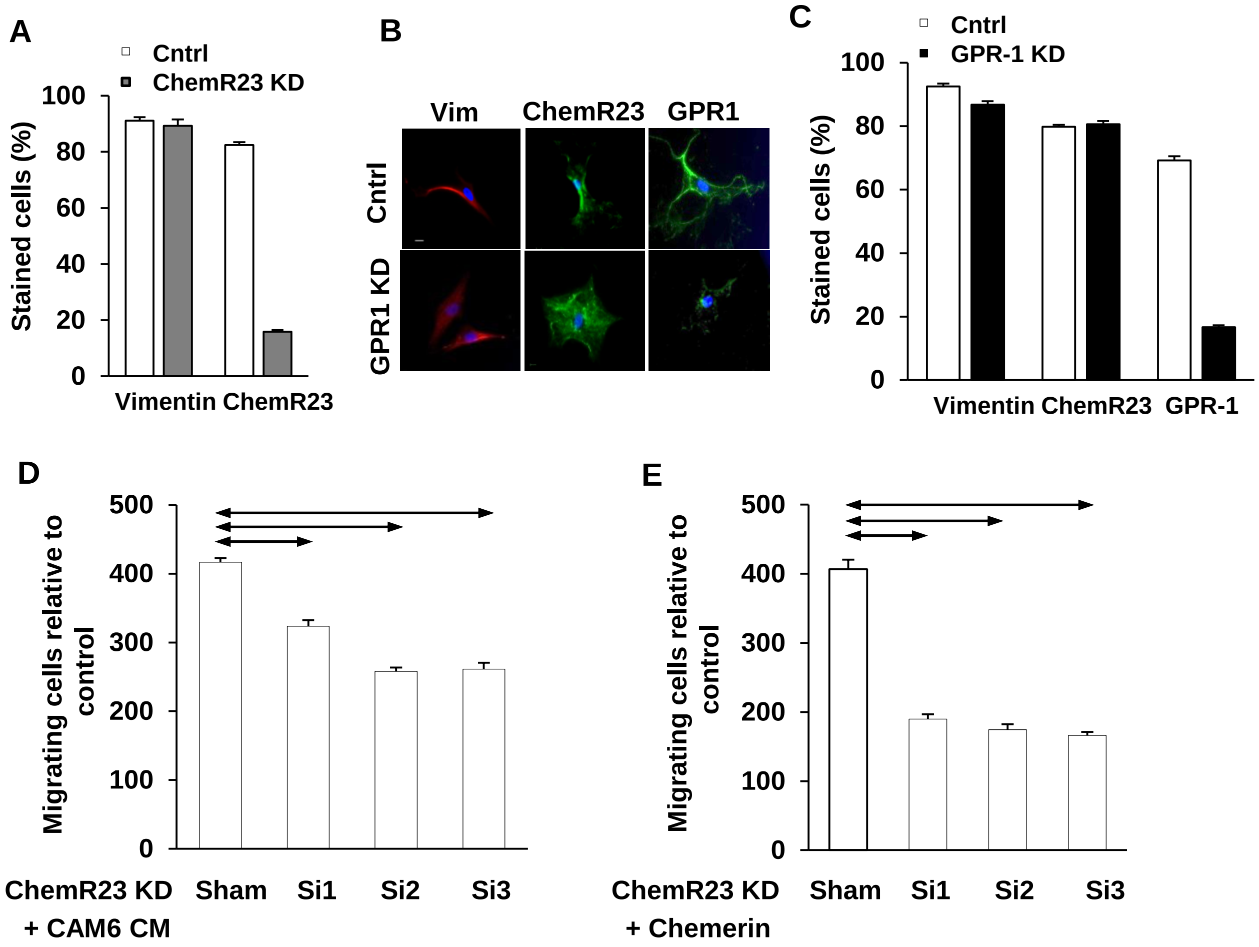

Supplementary Figure S7. SiRNA Knockdown of ChemR23. *A*, quantification of cells expressing ChemR23 after siRNA knockdown compared with vimentin as a negative control. *B,* Representative image from MSCs stained for GPR1, ChemR23 and vimentin after GPR1 siRNA knock down. *C,* quantification of cells expressing GPR-1 after GPR1 knockdown compared with vimentin and ChemR23 (negative controls). *D*,Boyden chamber migration assays of MSCs treated with CAM-CM after ChemR23 knockdown with three different siRNAs. *E*, Boyden chamber migration assays of MSCs treated with chemerin after ChemR23 knockdown with three different siRNAs, n=4-6.

Supplementary Figure S8.

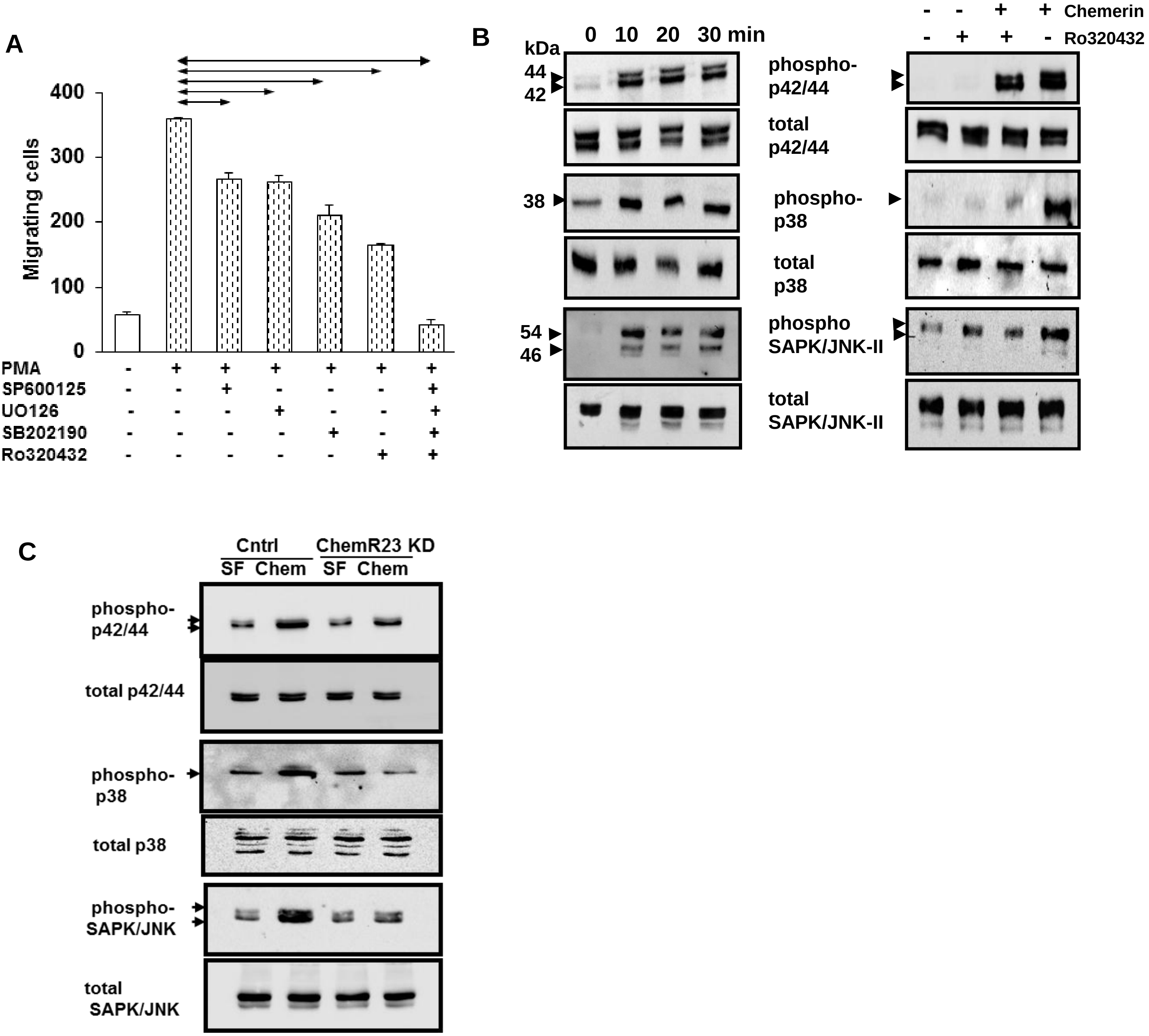

Supplementary Figure S8. MAP kinases mediate the action of PMA. *A,* PMA (100nM) stimulated MSC migration in Boyden chamber assays and was inhibited by SP600125 (JNK-II inhibitor, 50µM), U0126 (p42/44 inhibitor, 10µM), and SB202190 (p38 inhibitor, 3µM). *B,* Representative Western blots of phospho p42/44, p38 and JNK-II kinases from MSC cell extracts treated with PMA (left), Ro320432 (Ro, PKC inhibitor) and chemerin (100 ng/ml). *C,* Representative Western blot of phospho- and total p42/44 kinase, p38 kinase, and JNK-II expression in response to chemerin (100 ng/ml) in control MSCs and after chemR23 knock-down. Horizontal arrows mean $p<0.05$ by ANOVA, n= 4-6.

Supplementary Figure S9.

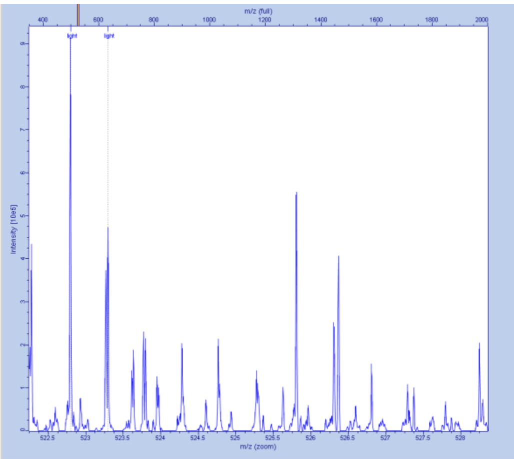

Sequence: LLCGLLAER

MIF

```
10         20         30         40         50         60
MPMFIVNTNV PRASVPDGFL SELTQQLAQA TGKPPQYIAV HVVPDQLMAF GGSSEPCALC
        70         80         90        100        110
SLHSIGKIGG AQNRSYSKLL CGLLAERLRI SPDRVYINYY DMNAANVGWN NSTFA
```

Sequence	Chemerin:Control mean ratio	Quantified in (out of 3 replicates)
LLCGLLAER	1.87	3
PMFIVNTNVPR	1.78	3

Supplementary Figure S9. Identification of MIF in MSC cell extracts using SILAC labelled cells treated with chemerin or not, and processed by LC-MS/MS. Top panel shows representative spectrum, center panel shows tryptic peptides identified (underlined), and lower panel shows relative abundance of two tryptic peptides in chemerin and control samples.

Supplementary Figure S10

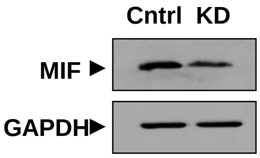

Supplementary Figure S10. Representative of Western blot analysis of MSC cell extract after MIF knock-down.
